# Genetic Variants Modulating CRIPTO Serum Levels Identified by Genome-Wide Association Study in Cilento Isolates

**DOI:** 10.1371/journal.pgen.1004976

**Published:** 2015-01-28

**Authors:** Daniela Ruggiero, Stefania Nappo, Teresa Nutile, Rossella Sorice, Francesco Talotta, Emilia Giorgio, Celine Bellenguez, Anne-Louise Leutenegger, Giovanna L. Liguori, Marina Ciullo

**Affiliations:** 1 Institute of Genetics and Biophysics A. Buzzati-Traverso, CNR, Naples, Italy; 2 Institut Pasteur de Lille, Lille, France; 3 Inserm, U744, Lille, France; 4 Université Lille-Nord de France, Lille, France; 5 Inserm, U946, Paris, France; 6 Université Paris Diderot, Sorbonne Paris Cité, IUH, UMR-S 946, Paris, France; Children’s Hospital of Philadelphia, UNITED STATES

## Abstract

*Cripto*, the founding member of the EGF-CFC genes, plays an essential role in embryo development and is involved in cancer progression. Cripto is a GPI-anchored protein that can interact with various components of multiple signaling pathways, such as TGF-β, Wnt and MAPK, driving different processes, among them epithelial-mesenchymal transition, cell proliferation, and stem cell renewal. Cripto protein can also be cleaved and released outside the cell in a soluble and still active form. *Cripto* is not significantly expressed in adult somatic tissues and its re-expression has been observed associated to pathological conditions, mainly cancer. Accordingly, CRIPTO has been detected at very low levels in the plasma of healthy volunteers, whereas its levels are significantly higher in patients with breast, colon or glioblastoma tumors. These data suggest that CRIPTO levels in human plasma or serum may have clinical significance. However, very little is known about the variability of serum levels of CRIPTO at a population level and the genetic contribution underlying this variability remains unknown. Here, we report the first genome-wide association study of CRIPTO serum levels in isolated populations (n = 1,054) from Cilento area in South Italy. The most associated SNPs (p-value<5*10-8) were all located on chromosome 3p22.1-3p21.3, in the *CRIPTO* gene region. Overall six CRIPTO associated loci were replicated in an independent sample (n = 535). Pathway analysis identified a main network including two other genes, besides *CRIPTO*, in the associated regions, involved in cell movement and proliferation. The replicated loci explain more than 87% of the CRIPTO variance, with 85% explained by the most associated SNP. Moreover, the functional analysis of the main associated locus identified a causal variant in the 5’UTR of *CRIPTO* gene which is able to strongly modulate *CRIPTO* expression through an AP-1-mediate transcriptional regulation.

## Author Summary

*Cripto* gene has a fundamental role in embryo development and is also involved in cancer. The protein is bound to the cell membrane through an anchor, that can be cleaved, causing the secretion of the protein, in a still active form. In the adult, CRIPTO is detected at very low levels in normal tissues and in the blood, while its increase in both tissues and blood is associated to pathological conditions, mainly cancer. As other GPI linked proteins such as the carcinoembryonic antigen (CEA), one of the most used tumor markers, CRIPTO is able to reach the bloodstream. Therefore, CRIPTO represents a new promising biomarker and potential therapeutic target, and blood CRIPTO levels might be associated to clinical features. Here we examined the variability of blood CRIPTO levels at a population level (population isolates from the Cilento region in South Italy) and we investigated the genetic architecture underlying this variability. We reported the association of common genetic variants with the levels of CRIPTO protein in the blood and we identified a main locus on chromosome 3 and additional five associated loci. Moreover, through functional analyses, we were able to uncover the mechanism responsible for the variation in CRIPTO levels, which is a regulation mediated by the transcriptional factor AP-1.

## Introduction

Cripto, also known as Teratocarcinoma-derived growth factor 1 (TDGF1), is the original member of the Epidermal Growth Factor-Cripto/Fibroblast growth factor Receptor Ligand 1/Cryptic (EGF-CFC) family of vertebrate proteins involved in embryo development [1–3]. Cripto has been isolated in human and mouse [3] and is a GPI-anchored membrane protein [4] that can function in both membrane anchored and soluble form [5,6]. It is involved in multiple signaling pathways, such as TGF-β, Wnt and MAPK/ERK pathways, it regulates essential steps in early embryogenesis and it is also involved in processes such as cell migration, epithelial-mesenchymal transition (EMT), stem cell maintenance, all processes which are implicated in cancerogenesis [7–14]. Cripto has also a role in angiogenesis, being able to enhance the proliferation, migration and invasion of human umbilical endothelial cells, to stimulate their differentiation into vascular-like structures in Matrigel and is also able to induce tumor neovascularization *in vivo* [13]. Cripto is expressed at very low levels in different adult tissue types and organs, among them a higher expression is detected in colon, skeletal muscle, heart, cortex of adrenal gland and cerebellum (http://www.biogps.org/ [15], http://www.proteinatlas.org/ [16]). Pathological re-expression is seen in a number of solid cancers. Most studies focused on the role of Cripto in breast and colorectal cancer [17–20], in inflammatory conditions and also in a macaque model of neuroAIDS [21]. Numerous studies have demonstrated correlation between high expression levels of CRIPTO and malignant transformation, tumor invasiveness, metastatic spreading and hence poor prognosis [17,22–27]. *In vitro* and *in vivo* functional studies confirm a strong involvement of Cripto in cancer development and indicate that its effect on tumorigenesis might strictly depend on the cellular context in which it acts [28–33].

Moreover, many data indicate Cripto as a promising target for cancer therapy. Adkins et coll. demonstrated that the block of Cripto signaling with an anti-CFC domain antibody determined a strong inhibition of tumor cell growth *in vivo* [34]. Ever since, different approaches based on the use of oligonucleotides, vaccines or antibodies have been successfully applied to target Cripto by inhibiting its activity and/or expression in tumors and in neurodegenerative diseases [35]. Cripto inhibition by different approaches always resulted in inhibition of cancer cell proliferation *in vitro* and of tumor growth *in vivo* [36].

CRIPTO has been also detected at very low levels in the plasma of healthy controls, whereas significantly higher concentrations have been found in patients with breast, colon or cerebral tumors [19,37]. In both studies, tumor tissues and patient-matched blood samples have been analyzed, showing that CRIPTO high levels in the plasma correspond to re-expression of CRIPTO in tumor tissues [19]. These data suggest that CRIPTO, as the carcinoembryonic antigen (CEA) (another GPI anchored protein and a widely used tumor marker), is able to reach the bloodstream, being potentially released by tumor cells through GPI anchor cleavage. All together these data indicate that CRIPTO represents both a promising biomarker and a valid target for therapeutic intervention in cancer and that blood CRIPTO levels in humans may have clinical significance.

The two studies on the measure of circulating CRIPTO in the plasma published so far were both conducted on a very small group of individuals: the study of Bianco and coworkers analysed 21 healthy donors, 33 patients with colon carcinoma and 75 patients with breast carcinoma or benign breast lesions while the study of Pilgaard and coworkers included 28 Glioblastoma Multiforme (GBM) patients, 4 low-grade glioma patients and 8 healthy controls. In the first case no statistically significant correlations were observed between CRIPTO plasma concentration and various clinicopathologic variables, including tumor size, lymph node involvement and proliferative index and the degree of positivity for CRIPTO in tumor sections [19]. In the second case higher levels of the protein correlated with a shorter overall survival [37].

In the present study, CRIPTO serum levels were measured in a population-based sample from three isolated villages of the Cilento area, South Italy and a very high heritability (>80%) was estimated underscoring the importance of its genetic determinants. We present the first genome-wide association study (GWAS) for CRIPTO protein levels aiming at identifying genetic variants associated with the levels of circulating protein in the serum and we report the functional characterization of the main associated locus.

## Results

Characteristics of the study participants are presented in Table 1. No difference was observed in the CRIPTO serum levels between men and women (p-value = 0.77 in the discovery; p-value = 0.65 in the replication). The median level of the protein was higher in the replication sample compared to the discovery sample (p-value<0.0001), because the proportion of individuals with CRIPTO serum levels below the threshold of assay quantification (< 1 pg/ml) is higher in the discovery (38%) than in the replication sample (27%) (see Methods). Significant heritability estimates were computed in both discovery (0.84, SE = 0.04) and replication (0.79, SE = 0.06) population samples (see Methods).

**Table 1:** Characteristics of the study subjects.

Cohort		Discovery	Replication
N° of Individuals		1054	535
Women %		56.5	54.2
Age (mean ± SD)		52.4 ± 19.8	52.9 ± 19.5
Genealogical Kinship (mean ± SD)		0.0032±0.0156	0.0083±0.0214
CRIPTO (pg/ml)			
All	median	27.5	184.4
	95% CI	21.3–34.3	133.2–211.7
	Range	0–1514.3	0–1253.2
Men	median	25.9	168.4
	95% CI	17.6–42.7	71.6–217.2
	Range	0–1514.3	0–1253.2
Women	median	29.0	188.9
	95% CI	19.6–35.6	117.7–221.4
	Range	0–1428.9	0–1174.2

### Loci associated with CRIPTO serum levels—discovery and replication

**Fig 1:**
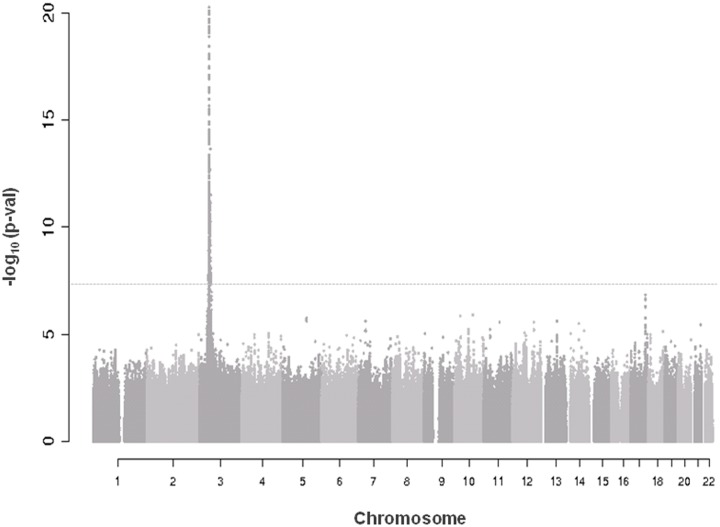
Manhattan plot of genome-wide association results in discovery analysis. Truncated Manhattan Plot showing -log_10_(p-values) for all SNPs of the CRIPTO discovery GWAS ordered by their chromosomal position. The dashed horizontal line represents the threshold for genome-wide significance (p-value<5*10^-8^). SNPs associated at the genome-wide significance level are all located on chromosome 3 (lowest p-value = 1.03*10^–159^).

A quantile-quantile plot for the 6,222,455 investigated autosomal SNPs in discovery GWAS revealed many more SNPs with low observed p-values than expected (S1 Fig.). 455 SNPs associated with CRIPTO serum concentration at p-value<5*10^–8^ in the discovery stage were located on chromosome 3 in a region spanning 6.4 Mb (3p22.11–3p21.31), with the most associated SNP (rs3806702, p-value = 1.03*10^–159^) located in the *CRIPTO* gene region. (Figs. 1 and S2). The C allele of this SNP was associated with higher levels of CRIPTO (CC = 673.8±29.0pg/ml; CT = 310.5±6.9pg/ml; TT = 46.7±4.4pg/ml) (Table 2). When running a conditional GWAS adjusting for the most associated SNP on chromosome 3, no loci remain associated at the genome-wide significance level, but 700 SNPs were associated with p-value<1*10^–4^ on the genome (S1 Table). Of those, linkage disequilibrium (LD)-based independent variants were defined if the pair-wise LD (r^2^) was less than 0.01 and if they were separated by at least 1 Mb. In this way, 95 LD-based independent loci were identified that included SNPs associated with CRIPTO serum concentration at a p-value<1*10^–4^. For each of the independent loci, the SNP with the lowest p-value was carried forward to replication. Criteria for replication were defined as a p-value<0.05 in the independent replication sample, the effect in the same direction between discovery and replication, and a p-value in the meta-analysis of discovery and replication samples lower than that obtained in the discovery sample (see Methods). Table 2 shows the summary results for the main association and the additional 5 replicated loci for CRIPTO serum levels. Regional association plots provide a detailed overview of those loci (S2 Fig.). The proportion of serum CRIPTO variance explained by the replicated loci in the discovery sample was 87.0%, with 84.9% explained by rs3806702 alone and 2.1% explained by the remaining 5 associated SNPs. Similar results were obtained in the replication sample, with 87.1% of CRIPTO variance explained by the 6 associated variants.

**Table 2:** Association results of replicated SNPs.

						Discovery analysis				Replication analysis				Meta-analysis		
Chr	SNP	Closest gene	Position (Mb)	Rsq	effect allele	effect allele frequency	Effect	s.e. of Effect	p-value	effect allele frequency	Effect	s.e. of Effect	p-value	Effect	s.e. of Effect	p-value
3	rs3806702	CRIPTO	46.62	1	C	0.23	1.325	0.049	1.03E-159	0.33	1.174	0.062	6.30E-79	1.267	0.039	8.64E-236
2	rs6739316	TNS1	218.77	0.65	G	0.76	-0.108	0.027	9.03E-05	0.81	-0.116	0.052	2.52E-02	-0.109	0.025	8.80E-06
9	rs17087824	NTRK2	87.50	0.89	T	0.94	-0.180	0.042	2.27E-05	0.89	-0.119	0.054	2.69E-02	-0.157	0.034	3.58E-06
14	rs74062852	SLC8A3	70.68	0.88	C	0.88	0.139	0.032	1.56E-05	0.85	0.136	0.048	4.72E-03	0.138	0.027	3.61E-07
15	rs7168855	MYO5A	52.72	0.53	T	0.89	-0.163	0.041	7.49E-05	0.94	-0.275	0.101	6.55E-03	-0.179	0.039	3.73E-06
17	rs6503271	GAS7	9.83	0.42	G	0.66	0.141	0.031	4.63E-06	0.66	0.115	0.052	2.88E-02	0.135	0.027	6.06E-07

Abbreviations: Chr-Chromosome, MAF-Minor Allele Frequency, s.e.-standard error, Rsq-imputation quality estimate

In the first row, the association result of the rs3806702 in the initial GWAS is shown. In the rows (2–6), the association results of the 5 replicated SNPs in the conditional GWAS adjusted for the rs3806702 genotype are reported.

### Biological pathway analysis

**Fig 2:**
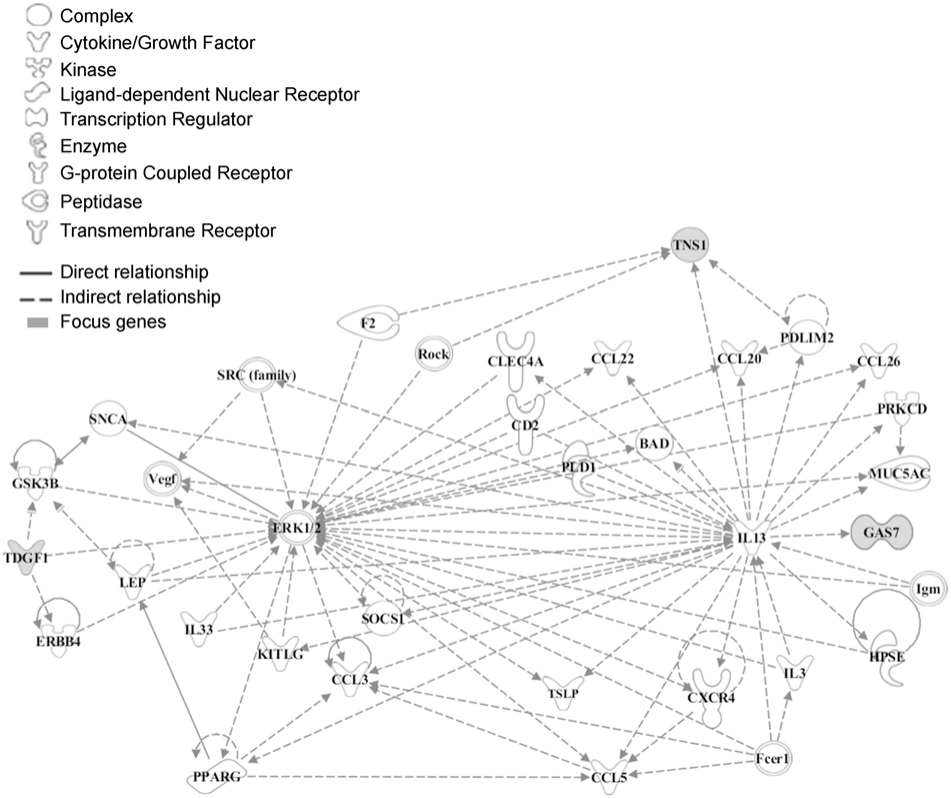
Functional analysis of the associated genes. The network was algorithmically constructed by Ingenuity Pathway Analysis (IPA) software on the basis of the functional and biological connectivity of genes. The network highlights the interconnections of 3 loci (marked in grey) identified from serum CRIPTO levels GWAS with a p-value = 1*10^–6^. *CRIPTO* gene is reported as *TDGF1*. Lines between genes represent known interactions and the nodes are displayed using various shapes that represent the functional class of the gene product (legend).

To explore the functional relationship between CRIPTO and the associated genes we used the Ingenuity Pathway Analysis software (IPA). *CRIPTO* gene and genes closest to the replicated SNPs were included in the analysis (see Methods). Those 10 seed genes were reported in the S2 Table. The IPA analysis identified a significant (p-value = 1*10^–6^) network of 35 molecules, 3 of which corresponded to CRIPTO associated loci (*CRIPTO* itself, *GAS-7* and *TNS-1*) (Fig. 2). Function categories assigned to the whole gene network by IPA were: “cell to cell signaling and interaction, hematological system development and function, and immune cell trafficking”. In depth, function annotation sub-categories significantly enriched in genes involved in the network and including *CRIPTO*, were: cell migration, proliferation of tumor cells, cell differentiation, and blood vessel development (S3 Table).

### Functional element analysis in associated loci

To identify functional elements in the associated loci, ENCODE data related to chromatin modifications and hypersensitivity DNAse elements were analyzed in 3 cell lines (NTERA-2, HepG2, H1-hESC) selected as expressing *CRIPTO* mRNA. Among the replicated SNPs and variants in LD with them (r^2^>0.5), 3 variants (rs3791936 in the intronic region of *TNS1*, rs112481213 in the 5’UTR of *CRIPTO*, and rs61117007 in the intronic region of *NTRK2*) were located in promoter or enhancer histone marks and 1 of those (rs112481213) was also located in DNA hypersensitivity elements in the selected cell lines, suggesting a potential functional role of those associated variants.

### Functional characterization of the associated rs112481213 variant

**Fig 3:**
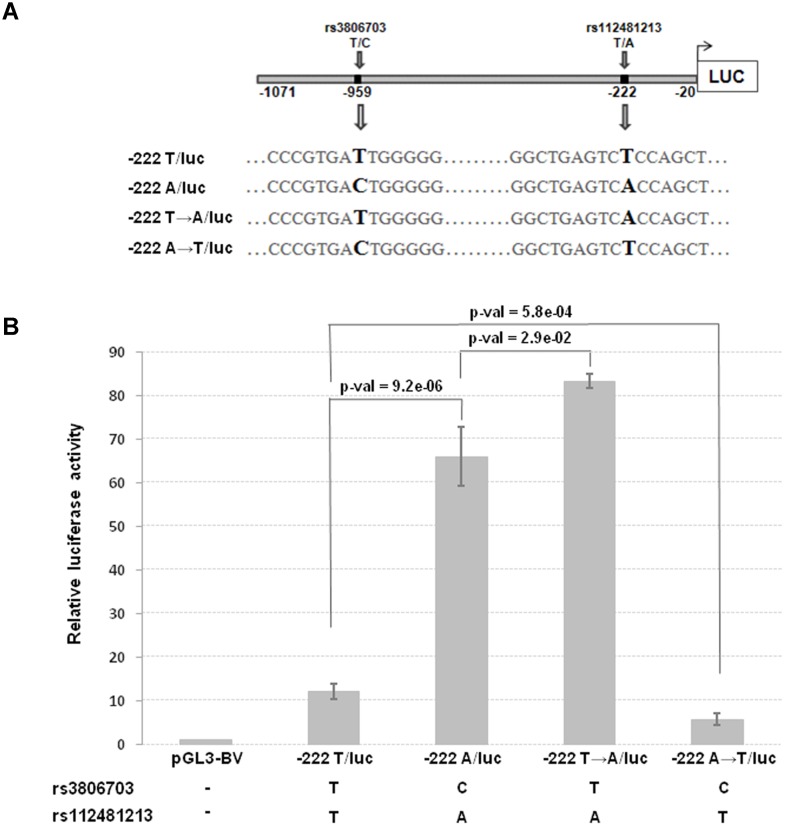
Transcriptional activity and schematic representation of reporter constructs. **(A)** The two constructs (222A/luc and-222T/luc) contain a 1,051 bp region including the 5’UTR and 342 bp upstream the transcription start site of *CRIPTO* and differ for the rs112481213 allele. The-222A→T/luc and 222T→A/luc constructs derive from a site-directed mutagenesis at the rs112481213 SNP position. **(B)** NTERA2 cell line was transfected with firefly luciferase reporter constructs carrying 1,051bp of the *CRIPTO* gene with either the A allele or the T allele at rs112481213. The rs112481213 A allele produced a 5-fold increase in luciferase activity compared to the T allele. Site-mutated constructs at the rs112481213 allowed to discriminate the effect of rs112481213 and rs3806703 on the transcription. Data are presented as fold-induction compared with promoter-less vector (pGL3-BV). Data are shown as mean±SD from five experiments.

The top 8 associated SNPs in the *CRIPTO* gene region were all included in a unique LD block (S3A–S3B Fig.). Among them the rs112481213 variant (LD with rs3806702, r^2^ = 0.99, p-value of association = 1.53*10^–158^), reported to be in a regulative region from ENCODE data (see the above paragraph), was also predicted to create an AP-1 binding site by bioinformatics analysis with MatInspector program and TRANSFAC database (S3C Fig.). To explore the possibility that this SNP, located in the 5’UTR of *CRIPTO* gene, might influence *CRIPTO* transcription we tested the SNP allele effect on the transcriptional activity in the NTERA2 teratocarcinoma cell line, expressing high levels of *CRIPTO* [38]. Two constructs, both containing a 1,051 bp region upstream the *CRIPTO* ATG start codon (including almost all the 5’UTR and also 342 bp upstream the transcription start site), but differing for the rs112481213 allele (-222A/luc and-222T/luc, respectively) were transfected in NTERA2 cells. Overall, 15 SNPs were included in that region. As one of those SNPs, the rs3806703 variant was in linkage disequilibrium with rs112481213 (r^2^ = 0.72), two additional constructs (-222T→A/luc, -222A→T/luc) were also produced by site-directed mutagenesis to discriminate the effect of each of the two variants on the transcription efficiency (Fig. 3A). The construct containing the rs112481213 A allele (-222A/luc) produced an about 5-fold increase (p-value = 9.2*10^–6^) of the luciferase activity compared to the construct containing the T allele (-222T/luc) indicating that rs112481213 SNP affects the transcription (Fig. 3B). Also, the luciferase activity was reported to high levels in the site-mutated-222T→A/luc construct demonstrating that the main effect on the transcription can be attributed to the rs112481213 variant. Interestingly, the activity produced by the-222T→A/luc is significantly higher than that observed for the-222A/luc (p-value = 2.9*10^–2^) and inverse results were obtained for-222A→T/luc and-222T/luc (p-value = 5.8*10^–4^), indicating that rs3806703 might also have an effect, although modest, on the transcription. A cooperative role of rs112481213 and rs3806703 was also statistically supported by an interaction model tested for the two variants (β_rs112481213_ = -1.18, SE_rs112481213_ = 0.04, β_rs3806703_ = 0.27, SE_rs3806703_ = 0.06, β_inter_ = -0.15, SE_inter_ = 0.03, p-value_inter_ = 7.08*10^–8^).

**Fig 4:**
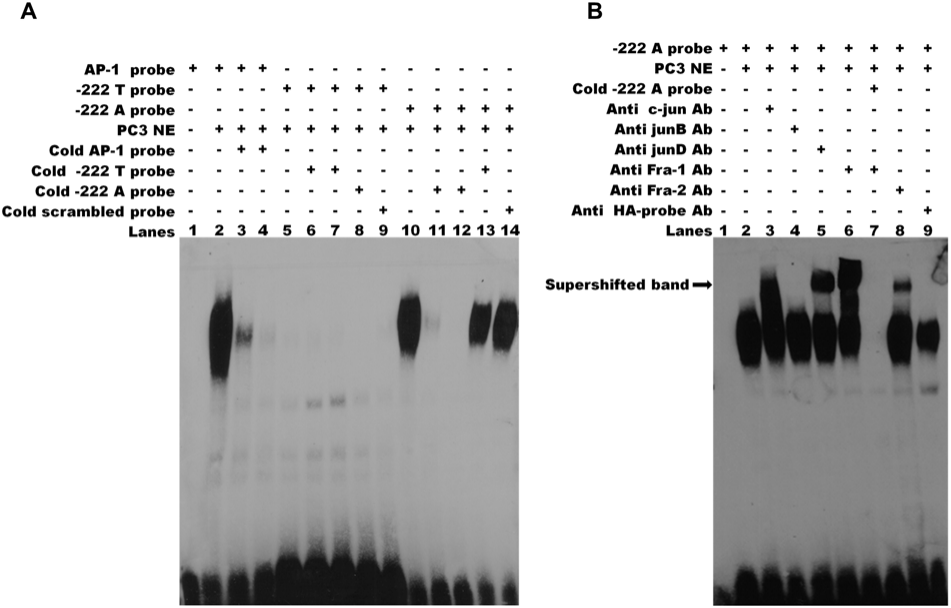
The rs112481213 variant alter the AP-1 binding site. **(A)** Electrophoretic mobility shift assay (EMSA) for AP-1 binding in nuclear extracts (NEs) of PC-3 cell line. The binding activities of NEs (lanes 2–14) were analyzed by EMSA using biotin-labeled AP-1 probe (lanes 2–4), -222 T probe (lanes 5–9) and-222 A probe (lanes 10–14). Competition was performed with 50 and 200 fold molar excess of the cold probe (lanes 3–4, 6–7, 11–12). Cross competition was performed using the cold probe carrying the alternative allele (lane 8 and 13) and the cold scrambled probe (lane 9 and lane 14) at 200 fold molar excess. No binding was detectable for-222 T probe (lanes 5–9). The DNA-protein complex of-222 A probe (lane 10) can be eliminated by 50 or 200 fold molar excess of cold-222 A probe (lanes 11, 12) but not by 200 fold molar excess of cold-222 T probe and cold scrambled probe. **(B)** Specificity of AP-1 binding activity. Supershift assay was carried out for-222 A probe using specific antibodies for the AP-1 components c-Jun (lane 3), JunB (lane 4), JunD (lane 5), Fra-1 (lane 6) and Fra-2 (lane 8). A supershifted DNA-protein-antibody complex (indicated by the arrow) is observed (lanes 3, 5, 6, 8). Lane 7 shows that the specific complexes were eliminated by addition of 200 fold molar excess of cold-222 A probe. No supershifted complex was observed adding HA-probe antibody used as negative control (lane 9). The results are representative of three independent determinations.

Therefore, the promoter activity data demonstrated that rs112481213 is a functional regulatory element of *CRIPTO* transcription which effect might be modulated by rs3806703. We next verified that the mechanism through which rs112481213 variant might influence *CRIPTO* transcription is the creation of an AP-1 consensus binding site, as predicted by bioinformatics analysis. Indeed, Electrophoretic Mobility Shift Assay (EMSA) using an AP-1 containing PC3 nuclear extract suggested that the A allele-containing oligonucleotide probe might robustly bind AP-1 complex while an oligonucleotide probe containing the T-allele weakly bound the complex (Fig. 4A). The AP-1 binding was confirmed by addition of specific antibodies for components of AP-1 complex, revealing a visibly supershifted band, representing a DNA-protein-antibody complex (Fig. 4B). All together, these data demonstrate the specificity of the observed DNA-protein interaction as well as a differential interaction of the AP-1 complex with the rs112481213 alleles.

## Discussion

*Cripto* is a typical example of an oncodevelopmental gene having key functions in early embryogenesis, and being re-expressed in the adult during tumorigenesis. Cripto is a GPI-anchored membrane protein, that can also be cleaved and released in the medium and is able to induce cellular proliferation, EMT, migration, and invasion, as well as to stimulate tumor angiogenesis both *in vitro* and *in vivo* [36]. Cripto promotes oncogenesis via modulation of TGF-β ligand signaling and through mechanisms that are independent of TGF-β ligands and their signaling receptors [39]. The effect of TGF-β ligands and Cripto on tumorigenesis is also dependent on the cellular context [33,39]. Interestingly, Cripto protein is an obligatory co-receptor for some TGF-β family members such as Nodal, enabling them to bind to Activin receptorial complexes and activate Smad cascade and is also able to antagonize the signaling of other members of the TGF-β family, (i.e., Activins and TGF-β), inhibiting their antioncogenic effect [34,35,40]. Moreover, Cripto acts via separate, non-overlapping mechanisms to enhance the canonical Wnt/β-catenin signaling pathway by binding to low-density lipoprotein receptor-related protein (LRP) 5 and LRP6 [41] and to activate ras/raf/MAPK and PI3K/Akt pathways via c-Src [28]. More recently, novel Cripto-interacting proteins, also involved in cancer, have been identified including the chaperonin glucose regulated protein-78 (Grp78) and Notch1 [39].

In humans, CRIPTO is expressed at very low levels in both normal tissues and plasma, while its expression was found increased in patients with cancer (also in both tumor tissues and plasma), suggesting that CRIPTO blood levels might have a great clinical relevance [19,37]. However in these two articles only a small number of healthy volunteers was analysed and no data on a general population sample were reported.

Our study is the first GWAS of circulating CRIPTO levels. It was undertaken in 1,589 individuals from three population isolates of the Cilento area, South Italy and represents the largest survey of CRIPTO measurement in a population-based sample. In our study, the variability of the circulating protein, according to the heritability estimation, was found to be highly determined by genetic factors. The GWAS identified the strongest association on chromosome 3 at rs3806702 located in the *CRIPTO* gene region. GWAS conditional to that variant showed that this locus represents the main genetic contribution to the modulation of CRIPTO in the serum. Indeed, 85% of the inherited component of circulating CRIPTO levels is explained by the rs3806702 variant. In accordance with this finding, all individuals with CRIPTO protein levels below the detection threshold (34% of the entire sample) were found homozygous for the T allele of the rs3806702 variant. The lower levels of CRIPTO in the discovery sample compared to the replication sample can also be explained by the difference in allele frequency (for T allele 0.77 and 0.67 respectively).

A LD block included rs3806702 as well as the top 7 associated SNPs in the *CRIPTO* gene region. Some of those were reported by bioinformatics analysis as potential candidates affecting transcription factor binding sites. Among those variants, rs112481213, located in the 5’UTR of *CRIPTO* gene, was identified by functional experiments as a causal SNP for *CRIPTO* transcriptional regulation. Regulation of *Cripto* expression during embryogenesis and tumorigenesis was still incompletely defined. So far different binding sites have been found in the promoter region of the *Cripto* gene: for Smad-proteins [42], the T-cell factor/lymphoid enhancer factor (Tcf/Lef) [43], the Hypoxia-Inducible Factor 1 (HIF-1) [44], the Nkx2–5 early cardiac transcription factor [45], and the orphan nuclear Liver Receptor Homolog-1 (LRH-1) [46]. HIF-1 and Nkx2–5 are able to transcriptionally activate *Cripto* during cardiac differentiation, HIF-1 also activates *CRIPTO* expression in human embryonal carcinoma cells, following hypoxic conditions [44,46]. Conversely, *CRIPTO* is directly repressed by the orphan nuclear receptor germ cell nuclear factor (GCNF) which binds to the promoter during retinoic acid-induced differentiation of human embryonic carcinoma cells and by the miR-15a/16 cluster which bind to the 3’UTR of *CRIPTO* mRNA [47,48]. However, this is the first time that an AP-1 transcriptional activation of *CRIPTO* has been described. We have indeed demonstrated that *CRIPTO* expression is regulated by AP-1 transcription factor and that this regulation depends on rs112481213 genotype. Transcriptional activity data also suggest that in addition to rs112481213, rs3806703, another SNP present in that region, may have a role in modulating the CRIPTO protein levels, possibly through the involvement of GATA binding transcriptional factors. In support to this hypothesis, a statistical interaction between rs112481213 and rs3806703 was also found. Due to the complexity of *Cripto* gene regulation and its dependency on the specific biological context, additional regulatory mechanisms might occur in the case of cancer-related cell dysfunction.

Further, five additional loci were associated to CRIPTO serum levels at p-values<1*10^–4^ independently from the main signal. Although these associations did not reach genome-wide significance in the discovery, likely because of lack of power of our study, they were replicated in an independent sample and might represent good candidate loci as modulators of the circulating CRIPTO. Moreover, three of the replicated variants were located in regions involved in regulative processes associated to chromatin accessibility.

A single network of 35 molecules including CRIPTO and two other associated loci, the growth-arrest-specific gene7 (*GAS-7*) and the Tensin1 (*TNS-1*), was identified by IPA analysis. The network included 30 genes implicated in cell migration, 18 genes involved in tumor cell proliferation, 25 genes in cell differentiation and, interestingly, 17 genes implicated in blood vessel development.

The analysis of CRIPTO associated loci showed that these are mainly linked to the MAPK/ERK signaling pathway with ERK1/2 as one of the principal players of the network. Aberrant regulation of MAPK cascades is known to strongly contribute to cancer and other human diseases.

Like *CRIPTO*, *TNS-1* and *GAS-7*, the other two genes in the associated regions present in the network, are both involved in breast and colon cancer [49–52]. TNS-1 binds to actin filaments [53] and serves as a link between signal transduction pathways and the actin cytoskeleton by forming a structural platform that regulates the assembly of focal adhesion components, phosphoproteins, and signaling molecules for processes such as cell migration [54]. *TNS-1* is expressed in normal tissues [55] while its expression is greatly reduced in human breast, prostate, head and neck squamous cell carcinomas, and melanoma suggesting a role as tumor suppressor [56] as well as in the maintenance of cell polarization, and the suppression of invasion that are involved in metastasis [57]. The TNS-1 phosphotyrosine binding (PTB) 1 domain binds the cytoplasmic tail of beta-integrin, presumed to be the basis for focal adhesion localization. Interestingly, overexpression of Cripto *in vitro* and *in vivo* has been associated with increased expression of fibronectin and various integrins and with increased activation of focal adhesion kinase [58].

Similarly to TNS-1, GAS-7 binds actin and participates in cytoskeleton dynamics, executing different functions in different cellular processes, such as vesicle trafficking, cell migration and morphological differentiation [59,60]. *GAS-7* hypermethylation has been found in breast and colon cancers whereas increased expression has been detected in medulloblastoma [61]. *GAS-7* expression is regulated by ERK signaling pathway [62], in which Cripto is also involved.

In the same CRIPTO associated locus on chromosome 17, besides *GAS-7*, is also located the G-protein-coupled receptor (*GLP-2R*) gene [63–65]. Interestingly, GLP-2R activation also induces ERK1/2 MAP kinase activation and is able to both stimulate the expression of the immediate early genes *c-Fos*, *c-Jun*, *JunB* and *Egr-1* and to activate AP1-driven gene transcription in a PKA-dependent manner [66,67].

Two other genes included in the associated loci, Myosin VA (*MYOVA*) gene on chromosome 15 and Neurotrophin tyrosine kinase receptor 2 (*NTRK2*) on chromosome 9, are both overexpressed in cancer. In particular, *MYOVA* is highly expressed in a number of highly metastatic cancer cell lines and metastatic colorectal cancer tissues and is able to interfere with metastatic capabilities by influencing cell migration. *MYOVA* expression is upregulated by the transcription factor Snail, one of the molecular switches for the EMT program involved in cancer metastasis [68]. *NTRK2* has been found frequently overexpressed in human cancers, including pancreatic and prostate carcinoma, Wilms’ tumor and neuroblastomas, particularly those with aggressive behavior and poor prognosis. As Cripto, NTRK2 activates both phosphatidylinositol-3-kinase (PI3K) and MAPK/ERK signaling.

In summary, our data showed that CRIPTO protein is measurable in the serum of the majority of individuals in a population-based sample. Further, we identified the largest genetic contribution to the CRIPTO variability and demonstrated that a functional variant located in the 5’UTR of *CRIPTO* gene is able to modulate *CRIPTO* expression through an AP-1-mediated transcriptional regulation. We also provided support for additional associated loci that will need to be confirmed in larger samples. Nevertheless, many of those associations converge in cancer phenotypes, mainly in cell movement and proliferation functions. As any association has been detected at these CRIPTO associated loci in large-scale cancer GWAS, further studies looking at CRIPTO variability in serum together with genotyping of the functional variant in specific cohorts of patients, focusing the analysis on specific cancer phenotypes, as metastasis formation, aggressiveness, prognosis, would be useful to better investigate possible associations between variants modulating CRIPTO protein levels and cancer features.

## Methods

### Population samples and CRIPTO measurement

The discovery sample includes 1,054 individuals recruited through a population-based sampling strategy in two small isolated villages of the Cilento region, South Italy (Gioi and Cardile) [69]. In silico replication was performed in additional 535 subjects from another village (Campora) of the same region [70]. The study design was approved by the ethics committee of Azienda Sanitaria Locale Napoli 1. The study was conducted according to the criteria set by the declaration of Helsinki and each subject signed an informed consent before participating to the study.

Blood samples were collected in the morning after the participants had been fasting for at least 12 h. Aliquots of serum were immediately prepared and stored at -80°C, and were subsequently used for the assessment of CRIPTO levels. CRIPTO (pg/ml) was measured using an enzyme-linked immunosorbent assay, according to the manufacturer’s instructions (DRG Instruments GmbH, Germany). An intra-assay coefficient of variation of the CRIPTO measure of 7.74% was obtained from 10 times measurements of 10 serum samples. Individuals with CRIPTO levels below the detection threshold were included in the study and a value of 0pg/ml was assigned to them.

Mann-Whitney U test was used to compare median CRIPTO serum levels among the samples.

A normal quantile transformation was applied to the trait and the transformed trait was used in all statistical analyses. The heritability of CRIPTO serum levels was estimated by SOLAR software [71] using extended genealogies of discovery and replication population samples and adjusting the phenotype for gender and age.

### GWAS and replication study

Genotyping was performed with 370K and Omniexpress Illumina chips, phasing and imputation were conducted separately by platform with the MaCH [72] and minimac (http://genome.sph.umich.edu/wiki/Minimac) software respectively, using 1000G v3 data as reference. SNP allele frequencies in Cilento samples versus 1000 Genomes reference allele frequencies for all genotyped SNPs were reported in the S4 Fig. Quality control filters applied before imputation were call rate >95% for SNPs and samples and minor allele frequency (MAF) >0.01. GWAS was carried out through a mixed model linear regression where the variance/covariance matrix is the genomic kinship to account for relatedness between individuals. Age and gender were used as covariates and an additive genetic model was considered. The analysis was performed with GenABEL package [73] for genotyped SNPs and ProbABEL [74] for imputed data. SNPs with imputation quality (Rsq in MACH) <0.4 or MAF <0.05 were excluded.

Conditional analysis was carried out in the discovery and replication samples adding the additive effect of rs3806702 as covariate in the association model.

To select linkage disequilibrium (LD)-based independent association signals among the CRIPTO associated SNPs, we conducted the clumping procedure implemented in PLINK [75] and picked the index SNPs with the most significant association p-value from each clumped association region based on the GWAS. The 1000G v3 genotypes were used as reference panel; the physical threshold for clumping was 1 Mb, and the r^2^ threshold for clumping was 0.01.

To assess evidence for replication, test-statistics of discovery and in silico replication samples were meta-analysed using a fixed effect model weighted by inverse variance, using Metal [76]. SNPs were considered replicated if the SNP p-value was <0.05 in the replication sample alone, the effect was in the same direction between discovery and replication, and the p-value in the meta-analysis was lower than in the discovery sample.

The percentage of the variance of the CRIPTO levels explained by the replicated SNPs was calculated both in the discovery and replication samples. Three linear mixed effects models were fitted, in which the CRIPTO was regressed, respectively, on: 1) gender and age (basic model); 2) gender, age, additive effect of a single SNP (single SNP model); 3) gender, age, additive effect of each of the replicated SNPs (multiple SNP model). The variance explained by each SNP was calculated as the difference between the variance explained by the single SNP model and that explained by the basic model. Similarly, the variance explained by the replicated SNPs all together was estimated as the difference between the variance explained by the multiple SNP model and that explained by the basic model. The *lmekin* function (R package), which uses the genomic kinship matrix to correct for relatedness between individuals, was applied.

To test for interaction between rs112481213 and rs3806703, a linear mixed eﬀect model, (including gender, age, additive effect of the two SNPs and the interaction between the two SNPs effects) implemented in the *lmekin* function (R package) was used. The variance inflation factor (VIF) was checked to be below ten to exclude collinearity problems [77].

### Pathway analysis

For each replicated SNP, seed genes were selected as located within a region of 100 kb upstream and 100 kb downstream the region delimited by SNPs in LD (r^2^>0.5) with it. For the main associated locus on chromosome 3, *CRIPTO* gene was included as seed. Overall, 12seed genes were analyzed with Ingenuity Pathway Analysis software (IPA, Ingenuity Systems, www.ingenuity.com) to explore the functional relationship between the proteins encoded by those genes. IPA tests a set of genes for enrichment in defined canonical pathways or functions and generates de novo networks of interacting genes or gene products. IPA computes a p-value, based on a Fisher’s exact test, that represents the likelihood of the core genes in a network and biological function being found together due to random chance. Direct and indirect interactions, a high confidence (experimentally observed or highly predicted) and a maximum size of 35 genes/proteins per network were used as parameters in the analysis.

### DNA functional element analysis

The associated loci were investigated for presence of chromatin histone marks and hypersensitive DNAse elements using data from ENCODE included in Haploreg software (http://www.broadinstitute.org/mammals/haploreg/) [78]. The replicated SNPs and variants in LD with them (r^2^>0.5) were analyzed. Cell lines where the CRIPTO mRNA was reported to be expressed in ENCODE database (www.genome.gov/encode/) were selected for the analysis.

### Constructs and transcription assays

A 1051-bp fragment upstream the ATG of the *CRIPTO* gene (position-1071/-20, RefSeq Gene NG_017049.1) was amplified by PCR from genomic DNA of individuals homozygous for either the A allele or the T allele of the rs112481213 at position-222 using as primers 5’-CGACGCGTCAAGCGGCACATCAGAGTC-3’ and 5’-GAAGATCTGAAAAGAGGCGTTAGCATCG-3’. The PCR products were digested with MluI/BglII and directionally cloned into the MluI and BglII sites of the luciferase reporter pGL3-basic vector (Promega, Madison, WI) to obtain-222A/luc and-222T/luc reporter constructs. The integrity of constructs was confirmed by DNA sequencing. Site-directed mutagenesis of both-222A/luc and-222T/luc constructs was performed using GeneArt Site-Directed Mutagenesis System (Invitrogen) according to the manufacturer’s protocol using as primers 5’-GAATCCCCGGAAAGGCTGAGTCACCAGCTCAAGGTCAAAACGTCC-3’ and 5’-GGACGTTTTGACCTTGAGCTGGTGACTCAGCCTTTCCGGGGATTC-3’ to perform the mutagenesis of-222T/luc (-222T→A/luc) and 5’-GAATCCCCGGAAAGGCTGAGTCTCCAGCTCAAGGTCAAAACGTCC-3’ and 5’-GGACGTTTTGACCTTGAGCTGGAGACTCAGCCTTTCCGGGGATTC-3’ for-222A/luc (-222A→T/luc). The constructs were then sequenced to confirm the sequence changes.

NTERA2 cells, cultured in Dulbecco’s modified Eagle’s medium F-12 (Gibco-Invitrogen) supplemented with 10% FBS at 37°C and 5% CO2, were transiently transfected using JetPRIME transfection reagent (PolyPlus Transfection) following the manufacturer’s protocol. Briefly, 0.5 μg of either pGL3-Basic vector, -222T/luc, -222A/luc, -222T→A/luc, -222A→T/luc together with 10 ng of Renilla luciferase reporter plasmid (Promega) were cotransfected. Luciferase activity was assayed at 48h using the Dual-Luciferase Reporter Assay System (Promega) according to the manufacturer’s protocol. Measurement of the firefly luciferase activity was normalized relative to the activity of the Renilla luciferase. Each construct was tested in triplicate in at least 3 independent experiments.

### Electrophoretic mobility shift and antibody supershift assays

All reactions included double-stranded, biotin-labeled oligonucleotide probe at 40fmol concentration. EMSAs were performed by using the LightShift Chemiluminescent EMSA kit (Pierce Biotechnology) according to the manufacturer’s protocol. PC3 cell line was used because of high expression of AP-1 components [79]. Nuclear extracts were prepared using Subcellular Protein Fractionation Kit for Cultured Cells (Pierce Biotechnology) according to the manufacturer’s protocol. Nuclear extracts (5μg) were incubated at room temperature either with the biotin-labeled probe alone or with the biotin-labeled probe and 50 or 200-fold molar excess unlabeled competitor probe for 20 min, before loading on a 4% nondenaturing acrylamide gel and subjected to autoradiography. The following double-stranded biotin-labeled oligonucleotides were used as probe: AP-1 control 5’-biotin-CGCTTGATGACTCAGCCGGAA-3’; -222T probe 5’-biotin-GAAAGGCTGAGTCTCCAGCTC-3’; -222A probe 5’-biotin-GAAAGGCTGAGTCACCAGCTC-3’. Also, a scrambled oligonucleotide (5’-GAAAGGCTTGACGACCAGCTC-3’) was used for competition at 200-fold molar excess.

Supershift assays were performed identically except for the addition of 3μg of antibody for 3h in ice before the addition of-222A probe. Antibodies used were anti-c-Jun, anti-JunB, anti-JunD, anti-Fra1 and anti-Fra2 [79] from Santa Cruz Biotechnology. HA-probe antibody against the influenza hemagglutinin (HA) protein (Santa Cruz Biotechnology), was used as negative control.

## Supporting Information

S1 Fig Quantile-Quantile (QQ) plot of CRIPTO level GWAS. For each variant tested, the observed -log10(p-value) (y-axis) is plotted against the expected -log10(p-value) under the null hypothesis (red line).
(DOCX)

S2 Fig Regional association plot of replicated loci. Regional association plots (panels A-F) show -log10(p-values) for all SNPs ordered by their chromosomal position within all regions of the replicated loci. For the rs3806702 the p-value of the discovery GWAS is reported, for the other SNPs p-values of the discovery conditional GWAS are reported. Each SNP is colored according to its correlation with the replicated SNP within the region as specified in the color scheme. Correlation structures correspond to hg19/1000 Genomes EUR Mar 2012. Plots were generated with LocusZoom [80]. The *CRIPTO* gene is reported as *TDGF1*.
(DOCX)

S3 Fig Genomic localization, linkage disequilibrium (LD) and transcription factor (TF) binding site analysis for the top 8 associated SNPs. **(A)** The genomic localization of the top 8 associated SNPs (red) respect to the *TDGF1* gene is shown using the GRCh38 primary assembly as reference sequence. The transcript (NM_003212.03) and the coding (CCDS2742.1) sequence are also shown. The functional SNP rs112481213 is underlined and reported in italic. The black rectangle indicates the region of 1.052 bp, upstream the ATG start codon, cloned in the luciferase reporter pGL3-basic vector. The rs3806703 SNP (orange and with a dashed line) is present in the cloned region but is not included in the top 8 associated SNPs (LD < 0.8 for rs112481213). **(B)** LD between the top 8 associated SNPs is reported. The analysis is carried out on the 1000 genome data (European panel) using the Haploview software. The distance in kb from the ATG start codon is shown on the left column. **(C)** MatInspector analysis. The results are reported only for the SNPs for which: 1) SNP alleles differently affect the TF biding site. In the schema, “Change” of the allele is-1 if the Alt allele disrupts the TF binding site and is 1 if the Alt allele creates a new TF binding site; 2) The value of the matrix similarity is higher than 0.9; 3) The position of the allele in the sequence logo shows an information content > 0.2 (y axis of the sequence logo). The black star represents the position of the SNP in the TF consesus binding site.
(DOCX)

S4 Fig Comparison of allele frequencies. Allele frequencies of all genotyped SNPs in the discovery or replication samples (y axis) are plotted against allele frequencies in 1000 Genomes European panel reference (x axis). RAF = Reference Allele Frequency.
(DOCX)

S1 Table Associated SNPs at 5*10–8<p-value<1*10–4 in the discovery conditional GWAS. (DOCX)

S2 Table Seed genes analyzed with Ingenuity Pathway Analysis software (IPA) in addition to CRIPTO (TDGF1) gene. (DOCX)

S3 Table Functional annotation sub-categories significantly enriched in genes involved in the network identified by IPA and including CRIPTO (TDGF1). (DOCX)
