# Supplementary figures and images for: Genetic Variants Modulating CRIPTO Serum Levels Identified by Genome-Wide Association Study in Cilento Isolates
Source: PLoS Genet. 2015 Jan 28;11(1):e1004976. doi: 10.1371/journal.pgen.1004976 (PMC4309561; doi:10.1371/journal.pgen.1004976)

**
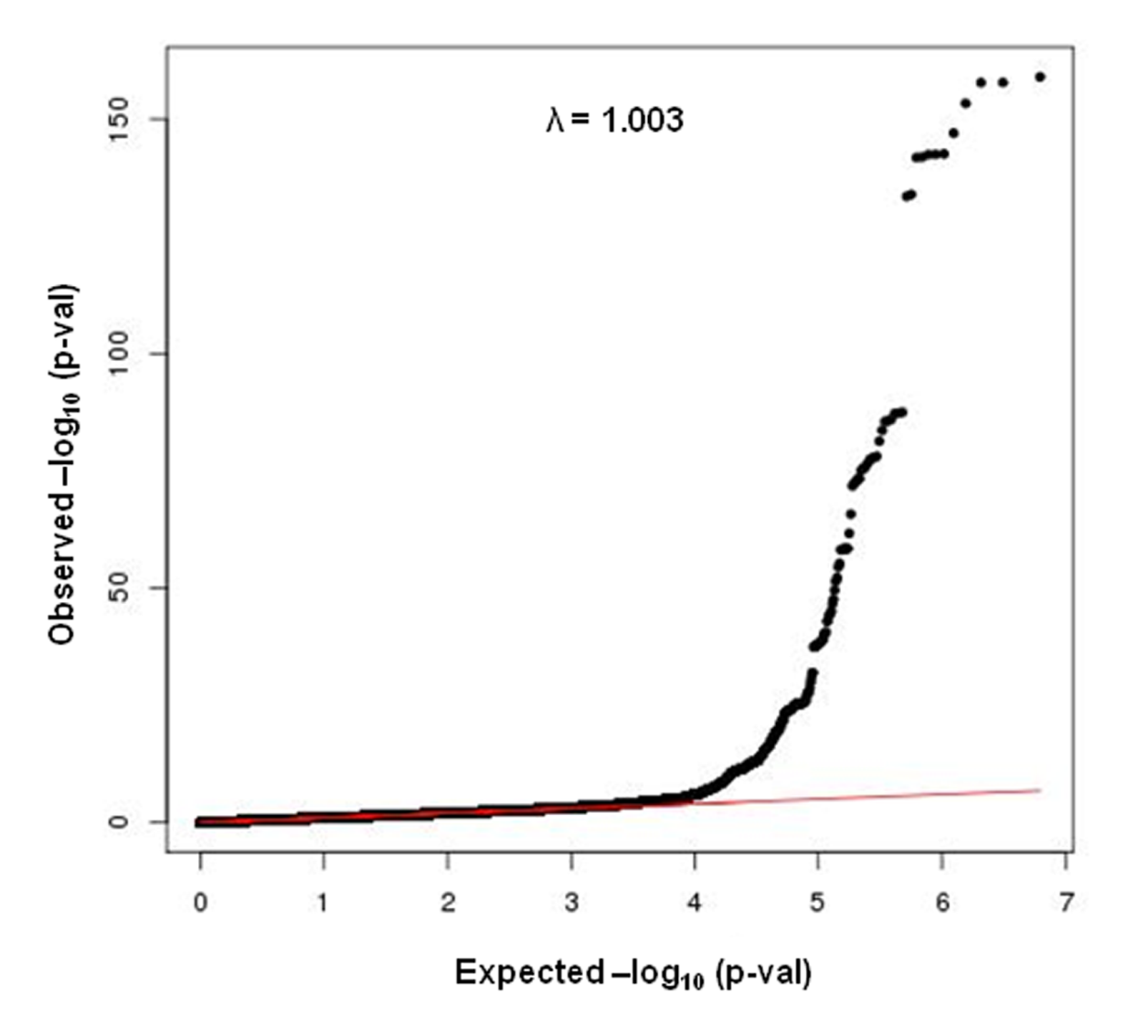
Figure S1.**

Supplement: S1 Fig — For each variant tested, the observed -log10(p-value) (y-axis) is plotted against the expected -log10(p-value) under the null hypothesis (red line). (DOCX) [file pgen.1004976.s001.docx]

**
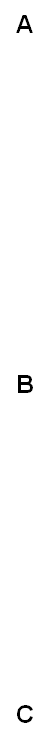
Figure S3**

Supplement: S3 Fig — (A) The genomic localization of the top 8 associated SNPs (red) respect to the TDGF1 gene is shown using the GRCh38 primary assembly as reference sequence. The transcript (NM_003212.03) and the coding (CCDS2742.1) sequence are also shown. The functional SNP rs112481213 is underlined and reported in italic. The black rectangle indicates the region of 1.052 bp, upstream the ATG start codon, cloned in the luciferase reporter pGL3-basic vector. The rs3806703 SNP (orange and with a dashed line) is present in the cloned region but is not included in the top 8 associated SNPs (LD < 0.8 for rs112481213). (B) LD between the top 8 associated SNPs is reported. The analysis is carried out on the 1000 genome data (European panel) using the Haploview software. The distance in kb from the ATG start codon is shown on the left column. (C) MatInspector analysis. The results are reported only for the SNPs for which: 1) SNP alleles differently affect the TF biding site. In the schema, “Change” of the allele is-1 if the Alt allele disrupts the TF binding site and is 1 if the Alt allele creates a new TF binding site; 2) The value of the matrix similarity is higher than 0.9; 3) The position of the allele in the sequence logo shows an information content > 0.2 (y axis of the sequence logo). The black star represents the position of the SNP in the TF consesus binding site. (DOCX) [file pgen.1004976.s003.docx]
