# Supplementary material for: Genetic Variants Modulating CRIPTO Serum Levels Identified by Genome-Wide Association Study in Cilento Isolates
Source: PLoS Genet. 2015 Jan 28;11(1):e1004976. doi: 10.1371/journal.pgen.1004976 (PMC4309561; doi:10.1371/journal.pgen.1004976)
Supplement: S2 Fig — Regional association plots (panels A-F) show -log10(p-values) for all SNPs ordered by their chromosomal position within all regions of the replicated loci. For the rs3806702 the p-value of the discovery GWAS is reported, for the other SNPs p-values of the discovery conditional GWAS are reported. Each SNP is colored according to its correlation with the replicated SNP within the region as specified in the color scheme. Correlation structures correspond to hg19/1000 Genomes EUR Mar 2012. Plots were generated with LocusZoom [80]. The CRIPTO gene is reported as TDGF1. (DOCX) [file pgen.1004976.s002.docx]

**Figure S2.**

A.

B.

C.

D.

E.

F.
