# Supplementary material for: Genetic Variants Modulating CRIPTO Serum Levels Identified by Genome-Wide Association Study in Cilento Isolates
Source: PLoS Genet. 2015 Jan 28;11(1):e1004976. doi: 10.1371/journal.pgen.1004976 (PMC4309561; doi:10.1371/journal.pgen.1004976)
Supplement: S1 Table — (DOCX) [file pgen.1004976.s005.docx]

**Table S1.**

| **SNP** | **Chr** | **position (Mb)** | **Rsq** | **effect allele** | **effect allele frequency** | **Effect** | **s.e. of Effect** | **p-value** |
| --- | --- | --- | --- | --- | --- | --- | --- | --- |
| rs4460663 | 1 | 7.19 | 1.00 | G | 0.41 | -0.0787 | 0.0202 | 9.51E-05 |
| rs7547331 | 1 | 18.09 | 0.96 | T | 0.67 | -0.0851 | 0.0216 | 8.00E-05 |
| rs11590632 | 1 | 18.09 | 0.92 | T | 0.67 | -0.0861 | 0.0220 | 8.86E-05 |
| rs76573519 | 1 | 103.58 | 0.97 | C | 0.93 | -0.1528 | 0.0391 | 9.25E-05 |
| rs75763405 | 1 | 103.58 | 0.97 | A | 0.93 | -0.1530 | 0.0391 | 9.08E-05 |
| rs149882644 | 1 | 103.59 | 0.96 | G | 0.93 | -0.1529 | 0.0391 | 9.32E-05 |
| rs11583146 | 1 | 103.59 | 0.98 | A | 0.93 | -0.1532 | 0.0391 | 9.05E-05 |
| rs11581384 | 1 | 103.59 | 0.98 | T | 0.93 | -0.1532 | 0.0391 | 9.06E-05 |
| rs6577357 | 1 | 103.59 | 0.98 | T | 0.93 | -0.1532 | 0.0392 | 9.20E-05 |
| rs78205563 | 1 | 103.59 | 0.98 | C | 0.93 | -0.1531 | 0.0392 | 9.24E-05 |
| rs61660832 | 1 | 103.59 | 0.98 | A | 0.93 | -0.1531 | 0.0392 | 9.28E-05 |
| rs6679075 | 1 | 103.59 | 0.98 | G | 0.93 | -0.1532 | 0.0392 | 9.39E-05 |
| rs11589450 | 1 | 103.60 | 0.99 | C | 0.93 | -0.1528 | 0.0392 | 9.83E-05 |
| rs189667291 | 1 | 103.60 | 0.94 | A | 0.93 | -0.1622 | 0.0399 | 4.81E-05 |
| rs113727867 | 1 | 103.60 | 0.94 | G | 0.93 | -0.1615 | 0.0399 | 5.20E-05 |
| rs56659384 | 1 | 103.63 | 0.96 | T | 0.92 | -0.1523 | 0.0369 | 3.73E-05 |
| chr1:103646918:GGC_G | 1 | 103.65 | 0.80 | R | 0.92 | -0.1610 | 0.0409 | 8.07E-05 |
| rs12065731 | 1 | 103.65 | 0.83 | T | 0.93 | -0.1700 | 0.0420 | 5.15E-05 |
| rs12060248 | 1 | 103.65 | 0.67 | G | 0.89 | -0.1578 | 0.0386 | 4.35E-05 |
| rs72690641 | 1 | 103.71 | 0.78 | A | 0.93 | -0.1791 | 0.0453 | 7.68E-05 |
| rs72690651 | 1 | 103.73 | 0.73 | A | 0.93 | -0.1866 | 0.0467 | 6.51E-05 |
| rs72690663 | 1 | 103.75 | 0.67 | G | 0.93 | -0.2007 | 0.0493 | 4.65E-05 |
| rs72690665 | 1 | 103.75 | 0.67 | T | 0.93 | -0.2017 | 0.0494 | 4.39E-05 |
| rs61141825 | 1 | 103.75 | 0.67 | C | 0.93 | -0.2019 | 0.0494 | 4.36E-05 |
| chr1:103751270:A_AT | 1 | 103.75 | 0.66 | R | 0.93 | -0.2031 | 0.0495 | 4.14E-05 |
| rs113985092 | 1 | 103.75 | 0.65 | G | 0.93 | -0.2024 | 0.0497 | 4.70E-05 |
| rs72690675 | 1 | 103.75 | 0.66 | G | 0.93 | -0.2027 | 0.0497 | 4.59E-05 |
| rs6674732 | 1 | 103.76 | 0.62 | G | 0.93 | -0.2002 | 0.0492 | 4.69E-05 |
| rs72690688 | 1 | 103.76 | 0.61 | C | 0.94 | -0.2151 | 0.0527 | 4.44E-05 |
| chr1:103758196:A_AT | 1 | 103.76 | 0.58 | R | 0.93 | -0.2021 | 0.0506 | 6.57E-05 |
| rs72690695 | 1 | 103.76 | 0.59 | T | 0.94 | -0.2124 | 0.0517 | 3.94E-05 |
| rs72692365 | 1 | 103.77 | 0.55 | G | 0.94 | -0.2197 | 0.0538 | 4.37E-05 |
| rs72692366 | 1 | 103.77 | 0.56 | G | 0.94 | -0.2231 | 0.0541 | 3.77E-05 |
| rs72692367 | 1 | 103.77 | 0.55 | A | 0.94 | -0.2205 | 0.0539 | 4.36E-05 |
| rs72692368 | 1 | 103.77 | 0.54 | T | 0.94 | -0.2234 | 0.0546 | 4.32E-05 |
| rs72692369 | 1 | 103.77 | 0.54 | T | 0.94 | -0.2235 | 0.0546 | 4.30E-05 |
| rs72692371 | 1 | 103.77 | 0.52 | T | 0.94 | -0.2373 | 0.0560 | 2.30E-05 |
| rs72692372 | 1 | 103.77 | 0.52 | C | 0.94 | -0.2374 | 0.0561 | 2.30E-05 |
| rs72692383 | 1 | 103.78 | 0.49 | A | 0.94 | -0.2509 | 0.0592 | 2.21E-05 |
| rs1356318 | 2 | 18.43 | 0.71 | A | 0.94 | -0.1897 | 0.0468 | 4.99E-05 |
| rs1356319 | 2 | 18.43 | 0.71 | T | 0.94 | -0.1899 | 0.0468 | 4.91E-05 |
| rs4832430 | 2 | 18.43 | 0.71 | A | 0.93 | -0.1903 | 0.0468 | 4.73E-05 |
| rs1452430 | 2 | 18.43 | 0.69 | G | 0.93 | -0.1785 | 0.0448 | 6.82E-05 |
| rs1452429 | 2 | 18.43 | 0.71 | T | 0.93 | -0.1790 | 0.0447 | 6.24E-05 |
| chr2:52714245:T_TG | 2 | 52.71 | 0.89 | R | 0.70 | -0.0958 | 0.0230 | 3.10E-05 |
| rs4605390 | 2 | 105.77 | 0.80 | C | 0.73 | 0.0930 | 0.0238 | 9.24E-05 |
| rs10166140 | 2 | 130.32 | 0.83 | T | 0.93 | -0.1575 | 0.0403 | 9.23E-05 |
| rs10174379 | 2 | 138.90 | 0.91 | C | 0.82 | 0.1050 | 0.0266 | 8.18E-05 |
| rs17706647 | 2 | 173.73 | 0.97 | C | 0.62 | -0.0803 | 0.0205 | 8.92E-05 |
| rs72946553 | 2 | 212.58 | 0.60 | C | 0.93 | -0.1814 | 0.0455 | 6.69E-05 |
| rs55771984 | 2 | 212.58 | 0.57 | T | 0.94 | -0.2051 | 0.0484 | 2.26E-05 |
| rs55861254 | 2 | 212.58 | 0.56 | G | 0.93 | -0.2013 | 0.0486 | 3.46E-05 |
| rs56186400 | 2 | 212.58 | 0.57 | C | 0.94 | -0.2131 | 0.0489 | 1.31E-05 |
| rs72946572 | 2 | 212.59 | 0.64 | C | 0.94 | -0.1953 | 0.0467 | 2.94E-05 |
| rs72948529 | 2 | 212.64 | 0.85 | A | 0.84 | -0.1213 | 0.0284 | 1.93E-05 |
| rs280626 | 2 | 215.56 | 1.00 | T | 0.22 | 0.0924 | 0.0234 | 8.02E-05 |
| rs6739316 | 2 | 218.77 | 0.65 | G | 0.76 | -0.1078 | 0.0275 | 9.03E-05 |
| chr2:227018112:G_GTA | 2 | 227.02 | 0.51 | R | 0.80 | -0.1331 | 0.0339 | 8.42E-05 |
| rs6759497 | 2 | 240.34 | 0.96 | A | 0.52 | 0.0814 | 0.0200 | 4.79E-05 |
| rs73010023 | 2 | 241.95 | 0.45 | G | 0.80 | -0.1483 | 0.0357 | 3.22E-05 |
| rs75019704 | 3 | 16.63 | 0.88 | G | 0.95 | 0.1887 | 0.0475 | 7.17E-05 |
| rs12487750 | 3 | 31.17 | 0.89 | A | 0.84 | -0.1079 | 0.0276 | 9.27E-05 |
| rs12495158 | 3 | 31.17 | 0.79 | G | 0.86 | -0.1219 | 0.0312 | 9.24E-05 |
| rs11925641 | 3 | 31.17 | 0.91 | C | 0.85 | -0.1113 | 0.0276 | 5.44E-05 |
| rs9814614 | 3 | 31.17 | 0.91 | T | 0.84 | -0.1110 | 0.0277 | 6.11E-05 |
| rs9810024 | 3 | 31.18 | 0.93 | C | 0.87 | -0.1154 | 0.0295 | 9.16E-05 |
| chr3:31177483:CT_C | 3 | 31.18 | 0.95 | R | 0.88 | -0.1253 | 0.0300 | 2.97E-05 |
| chr3:31177487:TG_T | 3 | 31.18 | 0.82 | R | 0.89 | -0.1404 | 0.0337 | 3.14E-05 |
| rs1485917 | 3 | 31.18 | 0.98 | C | 0.87 | -0.1244 | 0.0295 | 2.47E-05 |
| rs2221504 | 3 | 31.18 | 0.98 | A | 0.87 | -0.1245 | 0.0295 | 2.42E-05 |
| chr3:31182150:G_GA | 3 | 31.18 | 0.98 | R | 0.87 | -0.1244 | 0.0295 | 2.44E-05 |
| rs6784931 | 3 | 31.18 | 0.98 | T | 0.87 | -0.1244 | 0.0295 | 2.43E-05 |
| rs6772285 | 3 | 31.18 | 0.98 | G | 0.87 | -0.1244 | 0.0295 | 2.44E-05 |
| rs6785737 | 3 | 31.19 | 0.99 | G | 0.88 | -0.1250 | 0.0295 | 2.26E-05 |
| rs62233889 | 3 | 31.19 | 0.99 | C | 0.88 | -0.1258 | 0.0295 | 2.03E-05 |
| rs7434097 | 3 | 31.19 | 0.97 | T | 0.88 | -0.1256 | 0.0302 | 3.12E-05 |
| rs2135375 | 3 | 31.19 | 0.99 | T | 0.88 | -0.1254 | 0.0295 | 2.15E-05 |
| rs1121470 | 3 | 31.19 | 0.99 | C | 0.88 | -0.1253 | 0.0295 | 2.15E-05 |
| rs62233894 | 3 | 31.19 | 0.99 | G | 0.88 | -0.1255 | 0.0295 | 2.11E-05 |
| rs17027132 | 3 | 31.20 | 0.99 | G | 0.88 | -0.1257 | 0.0295 | 2.05E-05 |
| rs62233896 | 3 | 31.20 | 0.99 | T | 0.87 | -0.1270 | 0.0295 | 1.68E-05 |
| rs11918364 | 3 | 31.20 | 1.00 | A | 0.88 | -0.1261 | 0.0295 | 1.97E-05 |
| rs6767630 | 3 | 31.20 | 0.99 | C | 0.87 | -0.1247 | 0.0296 | 2.48E-05 |
| rs12637736 | 3 | 31.20 | 0.98 | G | 0.87 | -0.1233 | 0.0295 | 2.95E-05 |
| rs74790989 | 3 | 44.14 | 0.58 | A | 0.85 | -0.1387 | 0.0355 | 9.42E-05 |
| rs12633258 | 3 | 60.66 | 0.72 | G | 0.91 | -0.1611 | 0.0382 | 2.50E-05 |
| rs139364729 | 3 | 60.73 | 0.50 | A | 0.95 | -0.2036 | 0.0523 | 9.81E-05 |
| chr3:81668338:CT_C | 3 | 81.67 | 0.71 | D | 0.38 | 0.0965 | 0.0238 | 5.22E-05 |
| rs76957034 | 3 | 134.09 | 0.79 | T | 0.92 | -0.1629 | 0.0400 | 4.72E-05 |
| rs6799905 | 3 | 165.62 | 0.82 | A | 0.93 | -0.1615 | 0.0415 | 9.94E-05 |
| chr4:77357491:C_CT | 4 | 77.36 | 0.98 | R | 0.83 | -0.1044 | 0.0264 | 7.45E-05 |
| chr4:77357497:T_TAA | 4 | 77.36 | 0.98 | R | 0.83 | -0.1044 | 0.0264 | 7.46E-05 |
| rs189707263 | 4 | 77.36 | 0.83 | G | 0.86 | -0.1261 | 0.0306 | 3.74E-05 |
| rs11944802 | 4 | 77.36 | 1.00 | G | 0.82 | -0.1015 | 0.0259 | 8.73E-05 |
| rs10027712 | 4 | 77.36 | 1.00 | G | 0.82 | -0.1014 | 0.0258 | 8.67E-05 |
| chr4:77358426:ATG_A | 4 | 77.36 | 1.00 | R | 0.82 | -0.1023 | 0.0258 | 7.48E-05 |
| chr4:77358434:GTC_G | 4 | 77.36 | 0.99 | R | 0.83 | -0.1027 | 0.0261 | 8.28E-05 |
| rs11097355 | 4 | 77.36 | 1.00 | T | 0.82 | -0.1037 | 0.0258 | 5.84E-05 |
| rs11097356 | 4 | 77.36 | 1.00 | T | 0.18 | 0.1020 | 0.0258 | 7.93E-05 |
| rs11737407 | 4 | 77.36 | 1.00 | G | 0.82 | -0.1044 | 0.0258 | 5.33E-05 |
| rs66466813 | 4 | 77.36 | 1.00 | C | 0.82 | -0.1045 | 0.0258 | 5.26E-05 |
| rs28722184 | 4 | 77.36 | 1.00 | G | 0.82 | -0.1039 | 0.0258 | 5.67E-05 |
| rs66785096 | 4 | 77.36 | 1.00 | G | 0.82 | -0.1045 | 0.0259 | 5.27E-05 |
| rs11736785 | 4 | 77.36 | 0.99 | T | 0.82 | -0.1051 | 0.0260 | 5.18E-05 |
| rs11723217 | 4 | 77.36 | 0.99 | G | 0.82 | -0.1051 | 0.0260 | 5.19E-05 |
| rs66506142 | 4 | 77.36 | 0.96 | A | 0.93 | -0.1581 | 0.0383 | 3.61E-05 |
| rs113450947 | 4 | 77.37 | 0.96 | A | 0.93 | -0.1589 | 0.0382 | 3.10E-05 |
| rs66837396 | 4 | 77.37 | 0.96 | G | 0.93 | -0.1590 | 0.0381 | 3.09E-05 |
| rs67436027 | 4 | 77.37 | 0.96 | G | 0.93 | -0.1590 | 0.0381 | 3.09E-05 |
| rs17001966 | 4 | 77.37 | 0.93 | C | 0.92 | -0.1583 | 0.0382 | 3.39E-05 |
| rs17001970 | 4 | 77.37 | 0.95 | C | 0.92 | -0.1582 | 0.0381 | 3.34E-05 |
| rs67822548 | 4 | 77.37 | 0.96 | C | 0.93 | -0.1589 | 0.0382 | 3.13E-05 |
| chr4:77370392:T_TG | 4 | 77.37 | 0.95 | R | 0.92 | -0.1580 | 0.0381 | 3.44E-05 |
| rs66493323 | 4 | 77.38 | 0.95 | T | 0.92 | -0.1552 | 0.0382 | 4.92E-05 |
| rs66968407 | 4 | 77.38 | 0.95 | C | 0.92 | -0.1540 | 0.0382 | 5.60E-05 |
| rs72864633 | 4 | 77.38 | 0.94 | G | 0.92 | -0.1508 | 0.0380 | 7.36E-05 |
| rs72657863 | 4 | 77.38 | 0.95 | A | 0.92 | -0.1512 | 0.0382 | 7.47E-05 |
| rs116610528 | 4 | 77.39 | 0.95 | A | 0.92 | -0.1500 | 0.0381 | 8.41E-05 |
| rs1506502 | 4 | 103.02 | 0.98 | A | 0.84 | -0.1091 | 0.0277 | 8.03E-05 |
| rs1506503 | 4 | 103.02 | 0.97 | T | 0.82 | -0.1113 | 0.0265 | 2.65E-05 |
| rs173218 | 4 | 103.02 | 0.98 | C | 0.83 | -0.1137 | 0.0267 | 2.09E-05 |
| rs238489 | 4 | 103.02 | 0.98 | T | 0.83 | -0.1135 | 0.0267 | 2.20E-05 |
| chr4:103027829:AC_A | 4 | 103.03 | 0.47 | D | 0.87 | -0.1620 | 0.0413 | 8.64E-05 |
| rs9996962 | 4 | 103.03 | 0.95 | G | 0.83 | -0.1108 | 0.0270 | 4.04E-05 |
| rs238441 | 4 | 103.03 | 0.94 | A | 0.83 | -0.1150 | 0.0273 | 2.54E-05 |
| rs238510 | 4 | 103.04 | 0.95 | G | 0.83 | -0.1095 | 0.0271 | 5.18E-05 |
| rs238512 | 4 | 103.04 | 0.95 | A | 0.83 | -0.1085 | 0.0270 | 5.92E-05 |
| rs238515 | 4 | 103.04 | 0.95 | G | 0.83 | -0.1062 | 0.0270 | 8.30E-05 |
| rs13141782 | 4 | 103.04 | 0.95 | G | 0.83 | -0.1077 | 0.0270 | 6.78E-05 |
| rs238472 | 4 | 103.04 | 0.95 | G | 0.83 | -0.1072 | 0.0270 | 7.29E-05 |
| rs238475 | 4 | 103.04 | 0.95 | A | 0.83 | -0.1069 | 0.0270 | 7.77E-05 |
| rs238477 | 4 | 103.04 | 0.94 | G | 0.82 | -0.1064 | 0.0271 | 8.44E-05 |
| rs190224 | 4 | 103.05 | 0.95 | G | 0.83 | -0.1061 | 0.0270 | 8.69E-05 |
| rs236758 | 4 | 103.06 | 0.91 | A | 0.83 | -0.1070 | 0.0274 | 9.52E-05 |
| rs66690583 | 4 | 161.48 | 0.95 | T | 0.82 | -0.1062 | 0.0267 | 6.93E-05 |
| rs17039779 | 4 | 161.49 | 0.98 | T | 0.82 | -0.1076 | 0.0262 | 3.87E-05 |
| rs60916829 | 4 | 161.49 | 0.97 | T | 0.82 | -0.1091 | 0.0262 | 3.11E-05 |
| rs66792372 | 4 | 161.49 | 0.98 | T | 0.82 | -0.1084 | 0.0261 | 3.35E-05 |
| rs116819642 | 4 | 161.49 | 0.98 | C | 0.82 | -0.1092 | 0.0262 | 3.01E-05 |
| rs4285046 | 4 | 161.50 | 0.97 | G | 0.82 | -0.1116 | 0.0264 | 2.38E-05 |
| rs188696303 | 4 | 161.50 | 1.00 | A | 0.82 | -0.1123 | 0.0261 | 1.65E-05 |
| rs12511322 | 4 | 161.50 | 1.00 | A | 0.18 | 0.1127 | 0.0261 | 1.55E-05 |
| rs11100299 | 4 | 161.50 | 0.96 | G | 0.83 | -0.1158 | 0.0270 | 1.72E-05 |
| rs184738312 | 4 | 161.51 | 0.99 | C | 0.82 | -0.1125 | 0.0263 | 1.85E-05 |
| rs139016786 | 4 | 161.51 | 0.94 | A | 0.83 | -0.1186 | 0.0274 | 1.55E-05 |
| rs58619521 | 4 | 161.51 | 0.98 | G | 0.82 | -0.1161 | 0.0265 | 1.18E-05 |
| rs115549532 | 4 | 161.51 | 0.93 | A | 0.82 | -0.1158 | 0.0268 | 1.57E-05 |
| chr4:161508047:TAA_T | 4 | 161.51 | 0.85 | R | 0.81 | -0.1125 | 0.0277 | 4.88E-05 |
| rs116106726 | 4 | 161.51 | 0.94 | A | 0.82 | -0.1151 | 0.0268 | 1.78E-05 |
| rs17039821 | 4 | 161.51 | 0.98 | A | 0.82 | -0.1102 | 0.0260 | 2.27E-05 |
| rs67253127 | 4 | 161.51 | 0.96 | C | 0.81 | -0.1087 | 0.0260 | 2.98E-05 |
| rs17039832 | 4 | 161.51 | 0.98 | G | 0.82 | -0.1102 | 0.0260 | 2.28E-05 |
| rs72699270 | 4 | 161.51 | 0.97 | A | 0.85 | 0.1128 | 0.0279 | 5.16E-05 |
| rs55637299 | 4 | 161.51 | 0.99 | T | 0.82 | -0.1132 | 0.0262 | 1.54E-05 |
| rs55718213 | 4 | 161.51 | 1.00 | G | 0.82 | -0.1131 | 0.0261 | 1.48E-05 |
| rs2350467 | 4 | 161.52 | 1.00 | G | 0.59 | -0.0826 | 0.0199 | 3.30E-05 |
| rs17039833 | 4 | 161.52 | 1.00 | C | 0.82 | -0.1131 | 0.0261 | 1.46E-05 |
| rs58439076 | 4 | 161.52 | 1.00 | T | 0.82 | -0.1131 | 0.0261 | 1.48E-05 |
| rs17039834 | 4 | 161.52 | 1.00 | T | 0.82 | -0.1130 | 0.0262 | 1.57E-05 |
| rs17357485 | 4 | 161.52 | 0.97 | G | 0.85 | 0.1129 | 0.0278 | 4.88E-05 |
| rs7688507 | 4 | 161.52 | 1.00 | C | 0.59 | -0.0828 | 0.0199 | 3.22E-05 |
| rs72699277 | 4 | 161.52 | 0.95 | C | 0.85 | 0.1167 | 0.0278 | 2.61E-05 |
| rs57773286 | 4 | 161.52 | 1.00 | T | 0.82 | -0.1131 | 0.0261 | 1.46E-05 |
| rs62328653 | 4 | 161.52 | 0.97 | T | 0.85 | 0.1129 | 0.0278 | 4.83E-05 |
| rs7675509 | 4 | 161.52 | 0.99 | G | 0.59 | -0.0820 | 0.0200 | 4.16E-05 |
| chr4:161519895:CAG_C | 4 | 161.52 | 0.97 | R | 0.85 | 0.1130 | 0.0278 | 4.81E-05 |
| rs72699282 | 4 | 161.52 | 0.97 | C | 0.85 | 0.1129 | 0.0278 | 4.83E-05 |
| rs62328655 | 4 | 161.52 | 0.97 | G | 0.85 | 0.1130 | 0.0278 | 4.82E-05 |
| rs17039849 | 4 | 161.52 | 0.99 | C | 0.82 | -0.1131 | 0.0261 | 1.51E-05 |
| rs17291749 | 4 | 161.52 | 0.95 | C | 0.85 | 0.1110 | 0.0281 | 7.68E-05 |
| rs7686898 | 4 | 161.53 | 0.99 | G | 0.59 | -0.0827 | 0.0199 | 3.33E-05 |
| rs62329760 | 4 | 161.53 | 0.96 | T | 0.85 | 0.1130 | 0.0278 | 4.94E-05 |
| rs1898326 | 4 | 161.53 | 0.97 | T | 0.82 | -0.1134 | 0.0262 | 1.51E-05 |
| rs62329761 | 4 | 161.53 | 0.96 | G | 0.85 | 0.1130 | 0.0278 | 4.94E-05 |
| rs62329762 | 4 | 161.53 | 0.95 | A | 0.85 | 0.1145 | 0.0282 | 4.85E-05 |
| rs62329763 | 4 | 161.53 | 0.96 | G | 0.85 | 0.1130 | 0.0278 | 4.94E-05 |
| rs62329764 | 4 | 161.53 | 0.96 | G | 0.85 | 0.1129 | 0.0279 | 5.03E-05 |
| rs17358077 | 4 | 161.54 | 0.96 | A | 0.85 | 0.1130 | 0.0279 | 4.99E-05 |
| rs57867381 | 4 | 161.54 | 0.96 | G | 0.82 | -0.1129 | 0.0263 | 1.71E-05 |
| rs28670056 | 4 | 161.54 | 0.97 | C | 0.58 | -0.0842 | 0.0199 | 2.34E-05 |
| rs62326967 | 4 | 161.54 | 0.95 | G | 0.85 | 0.1132 | 0.0279 | 5.03E-05 |
| rs4691685 | 4 | 161.54 | 0.97 | A | 0.58 | -0.0848 | 0.0199 | 2.14E-05 |
| rs62326969 | 4 | 161.54 | 0.95 | G | 0.85 | 0.1129 | 0.0279 | 5.30E-05 |
| rs28379978 | 4 | 161.54 | 0.96 | A | 0.58 | -0.0843 | 0.0200 | 2.41E-05 |
| rs12505067 | 4 | 161.54 | 0.96 | A | 0.82 | -0.1127 | 0.0263 | 1.88E-05 |
| rs62326970 | 4 | 161.54 | 0.94 | T | 0.85 | 0.1124 | 0.0280 | 5.85E-05 |
| rs72701008 | 4 | 161.55 | 0.93 | T | 0.86 | 0.1139 | 0.0283 | 5.68E-05 |
| rs72701010 | 4 | 161.55 | 0.96 | C | 0.82 | -0.1124 | 0.0263 | 1.90E-05 |
| rs62326972 | 4 | 161.55 | 0.94 | A | 0.85 | 0.1123 | 0.0280 | 5.99E-05 |
| rs2175986 | 4 | 161.55 | 0.96 | C | 0.82 | -0.1124 | 0.0264 | 2.04E-05 |
| rs72701013 | 4 | 161.55 | 0.94 | T | 0.85 | 0.1122 | 0.0280 | 6.03E-05 |
| rs113147606 | 4 | 161.55 | 0.94 | T | 0.85 | 0.1122 | 0.0280 | 6.03E-05 |
| rs11945623 | 4 | 161.55 | 0.97 | A | 0.57 | -0.0828 | 0.0199 | 3.06E-05 |
| rs62326975 | 4 | 161.55 | 0.93 | G | 0.86 | 0.1143 | 0.0284 | 5.56E-05 |
| rs12499256 | 4 | 161.56 | 0.97 | G | 0.82 | -0.1118 | 0.0263 | 2.14E-05 |
| rs12499262 | 4 | 161.56 | 0.97 | G | 0.82 | -0.1118 | 0.0263 | 2.18E-05 |
| rs62326977 | 4 | 161.56 | 0.94 | T | 0.85 | 0.1128 | 0.0280 | 5.70E-05 |
| rs62326978 | 4 | 161.56 | 0.94 | T | 0.85 | 0.1127 | 0.0280 | 5.80E-05 |
| rs17039895 | 4 | 161.56 | 0.94 | G | 0.85 | 0.1127 | 0.0280 | 5.79E-05 |
| chr4:161557743:AAT_A | 4 | 161.56 | 0.89 | R | 0.85 | 0.1115 | 0.0280 | 6.93E-05 |
| rs17039901 | 4 | 161.56 | 0.96 | G | 0.82 | -0.1069 | 0.0262 | 4.53E-05 |
| rs141205776 | 4 | 161.56 | 0.94 | G | 0.85 | 0.1128 | 0.0280 | 5.69E-05 |
| rs79695229 | 4 | 161.56 | 0.98 | T | 0.82 | -0.1113 | 0.0263 | 2.37E-05 |
| rs77219618 | 4 | 161.56 | 0.98 | A | 0.82 | -0.1112 | 0.0263 | 2.39E-05 |
| rs138653666 | 4 | 161.56 | 0.94 | C | 0.85 | 0.1127 | 0.0280 | 5.73E-05 |
| rs62326979 | 4 | 161.56 | 0.94 | C | 0.85 | 0.1127 | 0.0280 | 5.76E-05 |
| rs72701028 | 4 | 161.56 | 0.98 | C | 0.82 | -0.1108 | 0.0264 | 2.64E-05 |
| rs17039903 | 4 | 161.56 | 0.94 | C | 0.85 | 0.1127 | 0.0280 | 5.74E-05 |
| rs72701033 | 4 | 161.56 | 0.98 | C | 0.82 | -0.1107 | 0.0263 | 2.59E-05 |
| rs17292745 | 4 | 161.57 | 0.94 | A | 0.85 | 0.1130 | 0.0280 | 5.63E-05 |
| rs62326980 | 4 | 161.57 | 0.94 | G | 0.85 | 0.1130 | 0.0281 | 5.64E-05 |
| rs12513148 | 4 | 161.57 | 0.98 | A | 0.82 | -0.1106 | 0.0263 | 2.64E-05 |
| rs11100301 | 4 | 161.57 | 0.98 | T | 0.82 | -0.1105 | 0.0263 | 2.70E-05 |
| rs11100302 | 4 | 161.57 | 0.98 | T | 0.82 | -0.1105 | 0.0263 | 2.70E-05 |
| rs75746789 | 4 | 161.57 | 0.93 | G | 0.85 | 0.1138 | 0.0282 | 5.26E-05 |
| rs62326983 | 4 | 161.57 | 0.91 | G | 0.86 | 0.1142 | 0.0292 | 9.18E-05 |
| rs62326984 | 4 | 161.57 | 0.91 | C | 0.86 | 0.1143 | 0.0292 | 9.23E-05 |
| rs62326985 | 4 | 161.57 | 0.91 | C | 0.86 | 0.1143 | 0.0292 | 9.28E-05 |
| chr4:161573215:T_TAA | 4 | 161.57 | 0.93 | R | 0.82 | -0.1105 | 0.0266 | 3.25E-05 |
| rs62326986 | 4 | 161.57 | 0.90 | C | 0.86 | 0.1187 | 0.0297 | 6.33E-05 |
| rs62326987 | 4 | 161.57 | 0.90 | G | 0.87 | 0.1164 | 0.0297 | 8.94E-05 |
| rs4691687 | 4 | 161.57 | 0.91 | T | 0.87 | 0.1165 | 0.0298 | 9.02E-05 |
| rs4690989 | 4 | 161.58 | 0.91 | A | 0.87 | 0.1164 | 0.0298 | 9.14E-05 |
| rs4691688 | 4 | 161.58 | 0.91 | G | 0.87 | 0.1164 | 0.0298 | 9.20E-05 |
| rs1436944 | 4 | 161.58 | 0.96 | C | 0.82 | -0.1087 | 0.0265 | 4.08E-05 |
| rs12502779 | 4 | 161.58 | 0.96 | A | 0.82 | -0.1086 | 0.0265 | 4.10E-05 |
| rs17292999 | 4 | 161.58 | 0.91 | G | 0.87 | 0.1163 | 0.0298 | 9.51E-05 |
| rs62327009 | 4 | 161.58 | 0.90 | A | 0.87 | 0.1188 | 0.0302 | 8.39E-05 |
| rs57619622 | 4 | 161.58 | 0.96 | T | 0.83 | -0.1094 | 0.0267 | 4.24E-05 |
| rs11100303 | 4 | 161.58 | 0.96 | T | 0.82 | -0.1086 | 0.0265 | 4.13E-05 |
| rs56658486 | 4 | 161.58 | 0.96 | T | 0.82 | -0.1086 | 0.0265 | 4.14E-05 |
| rs1561196 | 4 | 161.58 | 0.96 | C | 0.82 | -0.1064 | 0.0265 | 6.03E-05 |
| rs1370163 | 4 | 161.58 | 0.95 | A | 0.82 | -0.1063 | 0.0266 | 6.43E-05 |
| rs6839521 | 4 | 161.58 | 0.95 | C | 0.82 | -0.1063 | 0.0266 | 6.46E-05 |
| rs6853299 | 4 | 161.58 | 0.94 | G | 0.82 | -0.1060 | 0.0266 | 6.82E-05 |
| rs72701060 | 4 | 161.59 | 0.94 | G | 0.82 | -0.1060 | 0.0266 | 6.92E-05 |
| rs2099559 | 4 | 161.59 | 0.94 | C | 0.82 | -0.1056 | 0.0266 | 7.33E-05 |
| rs6850251 | 4 | 161.59 | 0.93 | T | 0.82 | -0.1053 | 0.0267 | 7.87E-05 |
| rs11100304 | 4 | 161.59 | 0.93 | T | 0.82 | -0.1051 | 0.0267 | 8.21E-05 |
| rs11933274 | 4 | 161.59 | 0.61 | T | 0.63 | -0.1056 | 0.0258 | 4.17E-05 |
| rs7674161 | 4 | 186.77 | 0.86 | C | 0.74 | -0.0986 | 0.0250 | 7.93E-05 |
| rs12654104 | 5 | 1.96 | 0.81 | T | 0.92 | -0.1627 | 0.0396 | 3.92E-05 |
| rs1858428 | 5 | 9.44 | 1.00 | A | 0.43 | -0.0864 | 0.0205 | 2.54E-05 |
| rs11133635 | 5 | 10.96 | 0.81 | G | 0.81 | -0.1075 | 0.0261 | 3.89E-05 |
| rs337132 | 5 | 122.44 | 0.98 | A | 0.82 | -0.1085 | 0.0263 | 3.77E-05 |
| rs335189 | 5 | 122.45 | 1.00 | C | 0.70 | -0.0910 | 0.0222 | 4.33E-05 |
| chr5:122487420:T_TC | 5 | 122.49 | 0.97 | I | 0.77 | -0.0997 | 0.0249 | 6.12E-05 |
| rs13197599 | 6 | 20.37 | 0.77 | A | 0.86 | 0.1277 | 0.0326 | 9.01E-05 |
| chr6:20404945:AG_A | 6 | 20.40 | 0.97 | R | 0.82 | 0.1020 | 0.0257 | 7.39E-05 |
| chr6:20408561:AGT_A | 6 | 20.41 | 0.98 | R | 0.82 | 0.1020 | 0.0257 | 7.06E-05 |
| rs4712494 | 6 | 20.41 | 0.98 | T | 0.82 | 0.1023 | 0.0256 | 6.47E-05 |
| chr6:23943702:A_ACT | 6 | 23.94 | 0.64 | I | 0.93 | -0.1793 | 0.0439 | 4.52E-05 |
| rs7776096 | 6 | 39.75 | 0.63 | A | 0.79 | -0.1184 | 0.0295 | 5.90E-05 |
| rs2857506 | 6 | 50.80 | 0.97 | G | 0.91 | -0.1399 | 0.0354 | 7.55E-05 |
| chr6:50800453:C_CA | 6 | 50.80 | 0.97 | R | 0.92 | -0.1390 | 0.0355 | 9.04E-05 |
| chr6:50801490:GAC_G | 6 | 50.80 | 0.96 | R | 0.91 | -0.1395 | 0.0353 | 7.93E-05 |
| rs75559219 | 6 | 67.74 | 0.96 | T | 0.86 | -0.1107 | 0.0284 | 9.49E-05 |
| rs7775432 | 6 | 77.31 | 0.83 | A | 0.67 | -0.0950 | 0.0226 | 2.63E-05 |
| rs72929723 | 6 | 92.21 | 0.90 | A | 0.88 | 0.1348 | 0.0320 | 2.59E-05 |
| rs56049558 | 6 | 92.21 | 0.91 | T | 0.88 | 0.1339 | 0.0318 | 2.56E-05 |
| rs7746740 | 6 | 92.22 | 0.90 | A | 0.88 | 0.1365 | 0.0316 | 1.55E-05 |
| rs113059453 | 6 | 92.22 | 0.86 | A | 0.89 | 0.1352 | 0.0340 | 7.11E-05 |
| rs72929738 | 6 | 92.22 | 0.95 | T | 0.88 | 0.1337 | 0.0312 | 1.78E-05 |
| rs72929739 | 6 | 92.22 | 0.96 | A | 0.89 | 0.1319 | 0.0312 | 2.35E-05 |
| rs17554698 | 6 | 92.22 | 0.95 | C | 0.88 | 0.1337 | 0.0312 | 1.78E-05 |
| rs12529119 | 6 | 92.22 | 0.95 | G | 0.88 | 0.1335 | 0.0311 | 1.81E-05 |
| rs17554782 | 6 | 92.23 | 0.95 | C | 0.88 | 0.1333 | 0.0311 | 1.84E-05 |
| rs17554851 | 6 | 92.23 | 0.96 | G | 0.89 | 0.1314 | 0.0311 | 2.45E-05 |
| rs17554927 | 6 | 92.23 | 0.96 | A | 0.89 | 0.1315 | 0.0311 | 2.43E-05 |
| rs72929749 | 6 | 92.23 | 0.96 | A | 0.89 | 0.1315 | 0.0311 | 2.42E-05 |
| rs72929765 | 6 | 92.23 | 0.97 | C | 0.89 | 0.1310 | 0.0312 | 2.63E-05 |
| rs72929767 | 6 | 92.23 | 0.98 | G | 0.89 | 0.1296 | 0.0312 | 3.21E-05 |
| rs17555108 | 6 | 92.23 | 0.98 | G | 0.89 | 0.1293 | 0.0311 | 3.30E-05 |
| rs112898523 | 6 | 92.24 | 0.98 | T | 0.89 | 0.1295 | 0.0311 | 3.21E-05 |
| rs149403296 | 6 | 92.24 | 0.98 | C | 0.89 | 0.1309 | 0.0311 | 2.59E-05 |
| rs143309544 | 6 | 92.24 | 0.98 | G | 0.89 | 0.1298 | 0.0311 | 3.07E-05 |
| rs72929779 | 6 | 92.24 | 0.98 | A | 0.89 | 0.1294 | 0.0312 | 3.27E-05 |
| rs72929780 | 6 | 92.24 | 0.97 | T | 0.89 | 0.1294 | 0.0312 | 3.43E-05 |
| rs72929789 | 6 | 92.25 | 0.99 | C | 0.89 | 0.1283 | 0.0311 | 3.79E-05 |
| rs72913203 | 6 | 92.25 | 0.99 | C | 0.89 | 0.1283 | 0.0311 | 3.79E-05 |
| rs17555739 | 6 | 92.26 | 1.00 | G | 0.89 | 0.1276 | 0.0311 | 4.15E-05 |
| rs72913210 | 6 | 92.26 | 0.99 | A | 0.89 | 0.1248 | 0.0311 | 5.87E-05 |
| chr6:127177410:A_AT | 6 | 127.18 | 0.81 | R | 0.94 | -0.1820 | 0.0467 | 9.89E-05 |
| rs12672267 | 7 | 32.26 | 0.91 | G | 0.68 | 0.0876 | 0.0221 | 7.56E-05 |
| rs36091304 | 7 | 73.05 | 0.71 | T | 0.77 | -0.1002 | 0.0257 | 9.71E-05 |
| rs799169 | 7 | 73.05 | 0.71 | A | 0.69 | -0.1070 | 0.0235 | 5.47E-06 |
| rs11986865 | 8 | 22.64 | 0.70 | T | 0.93 | -0.1736 | 0.0408 | 2.12E-05 |
| rs11988441 | 8 | 25.03 | 0.99 | G | 0.94 | 0.1683 | 0.0423 | 6.96E-05 |
| rs57646049 | 8 | 25.05 | 0.97 | G | 0.94 | 0.1700 | 0.0424 | 6.16E-05 |
| rs17245857 | 8 | 26.12 | 0.86 | G | 0.62 | -0.0832 | 0.0214 | 9.94E-05 |
| rs75102866 | 8 | 35.97 | 0.41 | T | 0.90 | -0.1976 | 0.0496 | 6.78E-05 |
| chr8:39493520:G_GT | 8 | 39.49 | 0.72 | R | 0.93 | 0.1866 | 0.0453 | 3.75E-05 |
| rs59707583 | 8 | 39.91 | 0.84 | A | 0.69 | 0.0926 | 0.0221 | 2.81E-05 |
| rs12678264 | 8 | 39.91 | 0.84 | T | 0.69 | 0.0927 | 0.0221 | 2.81E-05 |
| chr8:39914213:A_AC | 8 | 39.91 | 0.84 | R | 0.69 | 0.0927 | 0.0221 | 2.82E-05 |
| rs7817096 | 8 | 64.92 | 0.88 | T | 0.94 | 0.1758 | 0.0445 | 7.82E-05 |
| chr8:64957195:TAGGG | 8 | 64.96 | 0.72 | R | 0.92 | 0.1688 | 0.0420 | 5.79E-05 |
| rs2587577 | 8 | 73.01 | 1.00 | A | 0.10 | 0.1403 | 0.0328 | 1.85E-05 |
| rs7005108 | 8 | 103.58 | 0.95 | G | 0.59 | -0.0861 | 0.0206 | 2.86E-05 |
| rs4257984 | 8 | 103.58 | 1.00 | T | 0.61 | -0.0798 | 0.0205 | 9.74E-05 |
| rs4276657 | 8 | 103.58 | 1.00 | G | 0.61 | -0.0798 | 0.0205 | 9.52E-05 |
| chr8:104122954:TAGG_ | 8 | 104.12 | 0.64 | R | 0.70 | -0.1053 | 0.0258 | 4.42E-05 |
| rs996200 | 8 | 104.22 | 0.72 | A | 0.94 | -0.1912 | 0.0488 | 8.99E-05 |
| rs324519 | 9 | 9.04 | 1.00 | T | 0.37 | 0.0794 | 0.0204 | 9.78E-05 |
| rs4744834 | 9 | 80.15 | 0.96 | G | 0.74 | 0.0883 | 0.0226 | 9.17E-05 |
| rs11793480 | 9 | 87.49 | 0.93 | C | 0.91 | -0.1431 | 0.0352 | 4.80E-05 |
| rs73476417 | 9 | 87.49 | 0.91 | T | 0.91 | -0.1434 | 0.0352 | 4.66E-05 |
| rs56960370 | 9 | 87.50 | 0.89 | T | 0.94 | -0.1774 | 0.0424 | 2.83E-05 |
| rs60906968 | 9 | 87.50 | 0.88 | G | 0.94 | -0.1779 | 0.0423 | 2.63E-05 |
| chr9:87497110:AAC_A | 9 | 87.50 | 0.55 | R | 0.87 | -0.1631 | 0.0401 | 4.81E-05 |
| rs11788168 | 9 | 87.50 | 0.89 | T | 0.94 | -0.1798 | 0.0425 | 2.34E-05 |
| rs17087824 | 9 | 87.50 | 0.89 | T | 0.94 | -0.1801 | 0.0425 | 2.27E-05 |
| chr9:90810077:C_CA | 9 | 90.81 | 0.72 | R | 0.80 | -0.1174 | 0.0290 | 5.12E-05 |
| rs75590681 | 9 | 90.84 | 0.87 | C | 0.88 | -0.1322 | 0.0326 | 4.99E-05 |
| rs17053979 | 9 | 90.84 | 0.88 | C | 0.88 | -0.1322 | 0.0326 | 4.97E-05 |
| rs77614932 | 9 | 90.85 | 0.89 | A | 0.88 | -0.1315 | 0.0324 | 4.83E-05 |
| rs17053983 | 9 | 90.85 | 0.89 | G | 0.88 | -0.1314 | 0.0323 | 4.84E-05 |
| rs77604951 | 9 | 90.85 | 0.90 | G | 0.88 | -0.1318 | 0.0323 | 4.59E-05 |
| rs76029147 | 9 | 90.85 | 0.90 | G | 0.88 | -0.1317 | 0.0323 | 4.63E-05 |
| rs17054000 | 9 | 90.85 | 0.90 | G | 0.88 | -0.1317 | 0.0323 | 4.66E-05 |
| chr9:90851862:C_CT | 9 | 90.85 | 0.90 | R | 0.88 | -0.1317 | 0.0323 | 4.66E-05 |
| rs17054004 | 9 | 90.85 | 0.90 | G | 0.88 | -0.1317 | 0.0323 | 4.65E-05 |
| rs7029462 | 9 | 119.68 | 0.98 | A | 0.87 | -0.1200 | 0.0292 | 3.90E-05 |
| rs7022341 | 9 | 124.89 | 0.93 | G | 0.77 | -0.0964 | 0.0234 | 3.72E-05 |
| rs877731 | 9 | 124.89 | 0.93 | T | 0.77 | -0.0930 | 0.0234 | 6.84E-05 |
| rs1109301 | 9 | 124.89 | 0.96 | C | 0.77 | -0.0928 | 0.0230 | 5.61E-05 |
| rs16911507 | 9 | 124.92 | 0.85 | G | 0.92 | -0.1430 | 0.0366 | 9.19E-05 |
| rs113878379 | 10 | 29.67 | 0.74 | C | 0.90 | 0.1622 | 0.0373 | 1.37E-05 |
| rs111376125 | 11 | 20.18 | 0.73 | C | 0.93 | 0.1675 | 0.0430 | 9.71E-05 |
| rs60330865 | 11 | 20.18 | 0.72 | A | 0.93 | 0.1686 | 0.0430 | 8.90E-05 |
| rs80216726 | 11 | 20.18 | 0.76 | T | 0.93 | 0.1761 | 0.0428 | 3.92E-05 |
| rs2403584 | 11 | 20.21 | 0.98 | T | 0.94 | 0.1569 | 0.0397 | 7.70E-05 |
| rs9634000 | 11 | 82.06 | 0.88 | G | 0.83 | -0.1191 | 0.0287 | 3.30E-05 |
| rs7115867 | 11 | 87.98 | 0.96 | C | 0.92 | -0.1486 | 0.0358 | 3.25E-05 |
| rs7115998 | 11 | 87.98 | 0.98 | C | 0.92 | -0.1486 | 0.0357 | 3.19E-05 |
| chr11:87985493:C_CT | 11 | 87.99 | 0.96 | R | 0.92 | -0.1485 | 0.0359 | 3.43E-05 |
| chr11:87985497:TACAC | 11 | 87.99 | 0.96 | R | 0.92 | -0.1486 | 0.0359 | 3.42E-05 |
| rs12804675 | 11 | 87.99 | 0.95 | C | 0.92 | -0.1490 | 0.0360 | 3.52E-05 |
| rs12790540 | 11 | 87.99 | 0.95 | A | 0.92 | -0.1490 | 0.0360 | 3.52E-05 |
| rs61901662 | 11 | 87.99 | 0.95 | T | 0.87 | -0.1181 | 0.0291 | 5.02E-05 |
| chr11:87990315:T_TTA | 11 | 87.99 | 0.76 | R | 0.91 | -0.1599 | 0.0375 | 1.99E-05 |
| rs12800681 | 11 | 87.99 | 0.85 | G | 0.92 | -0.1661 | 0.0388 | 1.88E-05 |
| rs1960703 | 11 | 87.99 | 0.89 | G | 0.93 | -0.1585 | 0.0388 | 4.39E-05 |
| rs61027720 | 11 | 87.99 | 0.88 | T | 0.93 | -0.1585 | 0.0389 | 4.50E-05 |
| rs12790083 | 11 | 87.99 | 0.89 | G | 0.93 | -0.1592 | 0.0390 | 4.47E-05 |
| rs35404378 | 11 | 87.99 | 0.89 | T | 0.93 | -0.1593 | 0.0391 | 4.63E-05 |
| rs7930145 | 11 | 88.00 | 0.88 | G | 0.88 | -0.1320 | 0.0318 | 3.25E-05 |
| rs34632072 | 11 | 88.00 | 0.88 | T | 0.93 | -0.1592 | 0.0395 | 5.65E-05 |
| rs7948930 | 11 | 88.00 | 0.87 | G | 0.93 | -0.1562 | 0.0395 | 7.78E-05 |
| rs10160699 | 11 | 95.43 | 0.62 | C | 0.82 | -0.1229 | 0.0303 | 4.96E-05 |
| rs11213133 | 11 | 97.76 | 0.97 | C | 0.57 | -0.0831 | 0.0211 | 8.37E-05 |
| rs147976261 | 12 | 8.68 | 0.93 | T | 0.72 | 0.0912 | 0.0232 | 8.64E-05 |
| rs7954950 | 12 | 8.68 | 0.94 | G | 0.68 | 0.0902 | 0.0222 | 4.88E-05 |
| rs55898717 | 12 | 8.68 | 0.95 | G | 0.68 | 0.0892 | 0.0222 | 5.74E-05 |
| rs112350665 | 12 | 8.69 | 0.95 | C | 0.68 | 0.0867 | 0.0221 | 9.03E-05 |
| rs1094609 | 12 | 75.05 | 0.47 | A | 0.53 | 0.1140 | 0.0272 | 2.78E-05 |
| rs938434 | 12 | 103.56 | 1.00 | G | 0.47 | -0.0867 | 0.0197 | 1.15E-05 |
| rs10466919 | 12 | 116.29 | 0.71 | T | 0.69 | 0.0951 | 0.0237 | 6.11E-05 |
| rs11059859 | 12 | 129.22 | 0.70 | G | 0.94 | -0.1898 | 0.0484 | 8.78E-05 |
| chr12:130002722:CAT_C | 12 | 130.00 | 0.99 | R | 0.66 | 0.0866 | 0.0204 | 2.27E-05 |
| rs12368099 | 12 | 130.00 | 0.99 | A | 0.66 | 0.0866 | 0.0204 | 2.26E-05 |
| rs3912893 | 12 | 130.00 | 1.00 | T | 0.66 | 0.0867 | 0.0204 | 2.16E-05 |
| rs12367259 | 12 | 130.00 | 1.00 | C | 0.66 | 0.0878 | 0.0204 | 1.72E-05 |
| rs3912369 | 12 | 130.01 | 1.00 | C | 0.66 | 0.0889 | 0.0205 | 1.41E-05 |
| rs4759956 | 12 | 130.01 | 0.99 | G | 0.63 | 0.0798 | 0.0204 | 9.25E-05 |
| rs73155239 | 12 | 130.01 | 0.99 | C | 0.66 | 0.0898 | 0.0205 | 1.21E-05 |
| rs10847892 | 12 | 130.01 | 0.98 | T | 0.66 | 0.0943 | 0.0206 | 4.74E-06 |
| rs11060396 | 12 | 130.01 | 0.97 | T | 0.65 | 0.0960 | 0.0206 | 3.10E-06 |
| rs11060397 | 12 | 130.01 | 0.96 | C | 0.65 | 0.0962 | 0.0208 | 3.58E-06 |
| rs10847900 | 12 | 130.03 | 0.95 | A | 0.52 | 0.0838 | 0.0203 | 3.51E-05 |
| rs4065486 | 13 | 21.04 | 0.89 | G | 0.90 | -0.1396 | 0.0352 | 7.15E-05 |
| rs9576092 | 13 | 32.00 | 0.93 | T | 0.85 | -0.1131 | 0.0282 | 6.24E-05 |
| rs73190948 | 13 | 47.07 | 0.75 | G | 0.94 | 0.1833 | 0.0446 | 3.93E-05 |
| rs73190952 | 13 | 47.08 | 0.75 | C | 0.94 | 0.1840 | 0.0446 | 3.66E-05 |
| chr13:47177145:ATAAT | 13 | 47.18 | 0.82 | R | 0.94 | 0.1724 | 0.0430 | 6.14E-05 |
| rs73190987 | 13 | 47.18 | 0.83 | C | 0.94 | 0.1722 | 0.0431 | 6.50E-05 |
| rs17319124 | 13 | 63.64 | 0.68 | T | 0.95 | -0.2189 | 0.0523 | 2.86E-05 |
| rs7145551 | 14 | 23.84 | 0.98 | C | 0.76 | 0.0948 | 0.0238 | 6.67E-05 |
| rs854407 | 14 | 25.20 | 0.93 | C | 0.85 | -0.1121 | 0.0284 | 7.74E-05 |
| rs1101637 | 14 | 25.21 | 0.96 | A | 0.85 | -0.1138 | 0.0286 | 7.09E-05 |
| rs712489 | 14 | 25.21 | 0.99 | C | 0.85 | -0.1151 | 0.0289 | 6.80E-05 |
| rs854311 | 14 | 25.22 | 0.96 | C | 0.86 | -0.1155 | 0.0296 | 9.29E-05 |
| rs147749929 | 14 | 25.22 | 0.97 | C | 0.86 | -0.1132 | 0.0290 | 9.48E-05 |
| chr14:25226802:AAT_A | 14 | 25.23 | 0.96 | R | 0.86 | -0.1162 | 0.0291 | 6.68E-05 |
| chr14:25226805:ATG_A | 14 | 25.23 | 0.96 | R | 0.86 | -0.1152 | 0.0291 | 7.59E-05 |
| rs854430 | 14 | 25.23 | 0.95 | C | 0.86 | -0.1134 | 0.0290 | 9.31E-05 |
| rs1101640 | 14 | 25.24 | 0.93 | A | 0.86 | -0.1135 | 0.0291 | 9.30E-05 |
| rs111587129 | 14 | 53.13 | 0.93 | G | 0.91 | 0.1411 | 0.0347 | 4.65E-05 |
| rs72684272 | 14 | 53.14 | 0.98 | T | 0.91 | 0.1385 | 0.0339 | 4.41E-05 |
| rs112161453 | 14 | 53.14 | 0.98 | C | 0.91 | 0.1413 | 0.0342 | 3.62E-05 |
| rs17125649 | 14 | 53.15 | 0.99 | A | 0.91 | 0.1368 | 0.0332 | 3.87E-05 |
| rs11157928 | 14 | 53.15 | 0.86 | T | 0.89 | 0.1349 | 0.0333 | 5.06E-05 |
| rs10483614 | 14 | 53.15 | 1.00 | C | 0.09 | -0.1396 | 0.0332 | 2.57E-05 |
| rs10483615 | 14 | 53.15 | 0.99 | A | 0.91 | 0.1380 | 0.0337 | 4.35E-05 |
| rs72684280 | 14 | 53.16 | 0.99 | C | 0.92 | 0.1482 | 0.0357 | 3.36E-05 |
| rs2357804 | 14 | 53.16 | 0.99 | C | 0.92 | 0.1476 | 0.0356 | 3.38E-05 |
| rs5017629 | 14 | 53.16 | 0.99 | T | 0.92 | 0.1483 | 0.0357 | 3.20E-05 |
| rs28478222 | 14 | 53.16 | 0.98 | A | 0.92 | 0.1477 | 0.0356 | 3.41E-05 |
| rs17107965 | 14 | 70.68 | 0.91 | G | 0.88 | 0.1302 | 0.0322 | 5.35E-05 |
| rs74062852 | 14 | 70.68 | 0.88 | C | 0.88 | 0.1391 | 0.0322 | 1.56E-05 |
| rs79159859 | 14 | 70.69 | 0.81 | C | 0.89 | 0.1378 | 0.0346 | 6.87E-05 |
| rs6574468 | 14 | 79.21 | 0.94 | A | 0.93 | -0.1615 | 0.0381 | 2.29E-05 |
| rs10150420 | 14 | 79.21 | 0.93 | T | 0.93 | -0.1579 | 0.0379 | 3.12E-05 |
| rs10142370 | 14 | 79.21 | 1.00 | A | 0.07 | 0.1555 | 0.0379 | 4.19E-05 |
| rs73317967 | 14 | 79.21 | 0.96 | C | 0.93 | -0.1612 | 0.0380 | 2.23E-05 |
| rs10151763 | 14 | 79.21 | 0.98 | G | 0.93 | -0.1564 | 0.0383 | 4.38E-05 |
| rs983501 | 14 | 85.05 | 0.93 | T | 0.59 | 0.0820 | 0.0204 | 5.75E-05 |
| chr14:85047285:CT_C | 14 | 85.05 | 0.92 | D | 0.59 | 0.0828 | 0.0206 | 5.60E-05 |
| rs1449102 | 14 | 85.05 | 0.93 | C | 0.59 | 0.0821 | 0.0204 | 5.60E-05 |
| rs10131132 | 14 | 85.05 | 0.93 | C | 0.59 | 0.0827 | 0.0204 | 5.01E-05 |
| rs979322 | 14 | 85.05 | 0.94 | C | 0.59 | 0.0826 | 0.0203 | 4.91E-05 |
| rs979323 | 14 | 85.05 | 0.93 | C | 0.59 | 0.0830 | 0.0203 | 4.56E-05 |
| rs1375448 | 14 | 85.05 | 0.94 | T | 0.59 | 0.0827 | 0.0203 | 4.87E-05 |
| rs10145762 | 14 | 85.05 | 0.93 | T | 0.59 | 0.0836 | 0.0204 | 4.16E-05 |
| rs12886820 | 14 | 85.05 | 0.93 | G | 0.59 | 0.0828 | 0.0204 | 4.78E-05 |
| rs12882856 | 14 | 85.05 | 0.94 | A | 0.47 | -0.0962 | 0.0203 | 2.24E-06 |
| rs12887175 | 14 | 85.05 | 0.93 | A | 0.59 | 0.0827 | 0.0204 | 5.14E-05 |
| rs10135177 | 14 | 85.05 | 0.94 | G | 0.53 | 0.0966 | 0.0203 | 1.95E-06 |
| rs12433873 | 14 | 85.05 | 0.93 | G | 0.59 | 0.0825 | 0.0204 | 5.35E-05 |
| rs10141189 | 14 | 85.05 | 0.90 | C | 0.60 | 0.0832 | 0.0208 | 6.46E-05 |
| rs10130659 | 14 | 85.05 | 0.93 | C | 0.59 | 0.0827 | 0.0204 | 5.24E-05 |
| rs10141909 | 14 | 85.05 | 0.94 | A | 0.59 | 0.0817 | 0.0204 | 6.08E-05 |
| rs10142013 | 14 | 85.05 | 0.94 | A | 0.59 | 0.0816 | 0.0204 | 6.19E-05 |
| rs10144447 | 14 | 85.05 | 0.94 | A | 0.59 | 0.0817 | 0.0204 | 6.10E-05 |
| chr14:85054403:TC_T | 14 | 85.05 | 0.90 | D | 0.58 | 0.0817 | 0.0207 | 8.16E-05 |
| chr14:85054825:T_TAA | 14 | 85.05 | 0.94 | I | 0.59 | 0.0825 | 0.0204 | 5.35E-05 |
| rs2998323 | 14 | 85.06 | 0.95 | A | 0.57 | 0.0970 | 0.0207 | 2.90E-06 |
| rs67853298 | 14 | 85.06 | 0.93 | A | 0.75 | 0.0913 | 0.0232 | 8.16E-05 |
| rs111991589 | 14 | 85.07 | 0.86 | G | 0.72 | 0.1109 | 0.0241 | 4.06E-06 |
| rs11620893 | 14 | 85.07 | 0.97 | A | 0.75 | 0.0885 | 0.0227 | 9.53E-05 |
| rs1037248 | 14 | 85.07 | 0.99 | G | 0.68 | 0.1005 | 0.0214 | 2.61E-06 |
| rs72692340 | 14 | 85.07 | 0.97 | A | 0.68 | 0.1013 | 0.0217 | 3.00E-06 |
| chr14:85070120:G_GT | 14 | 85.07 | 0.99 | R | 0.68 | 0.1006 | 0.0214 | 2.50E-06 |
| rs191049896 | 14 | 85.07 | 0.78 | C | 0.74 | 0.1277 | 0.0257 | 6.64E-07 |
| chr14:85070122:C_CT | 14 | 85.07 | 0.99 | R | 0.68 | 0.1006 | 0.0214 | 2.48E-06 |
| rs67271665 | 14 | 85.07 | 0.98 | G | 0.74 | 0.0883 | 0.0224 | 8.20E-05 |
| rs67627952 | 14 | 85.07 | 0.99 | C | 0.74 | 0.0882 | 0.0223 | 7.74E-05 |
| rs11848890 | 14 | 85.07 | 0.96 | A | 0.70 | 0.1024 | 0.0220 | 3.13E-06 |
| rs1449114 | 14 | 85.07 | 1.00 | G | 0.32 | -0.1045 | 0.0214 | 1.10E-06 |
| rs72692347 | 14 | 85.07 | 0.98 | A | 0.74 | 0.0914 | 0.0224 | 4.67E-05 |
| rs3008506 | 14 | 85.08 | 0.99 | A | 0.56 | 0.0986 | 0.0205 | 1.48E-06 |
| rs12050291 | 14 | 85.08 | 0.97 | G | 0.74 | 0.0894 | 0.0227 | 8.06E-05 |
| rs72692350 | 14 | 85.08 | 0.97 | G | 0.74 | 0.0906 | 0.0227 | 6.39E-05 |
| rs58516905 | 14 | 85.08 | 0.98 | G | 0.68 | 0.1020 | 0.0215 | 2.16E-06 |
| rs2998294 | 14 | 85.08 | 0.99 | C | 0.56 | 0.1011 | 0.0206 | 8.95E-07 |
| rs11159663 | 14 | 85.08 | 0.98 | T | 0.68 | 0.1020 | 0.0215 | 2.17E-06 |
| chr14:85080544:TA_T | 14 | 85.08 | 0.93 | R | 0.67 | 0.0978 | 0.0222 | 1.02E-05 |
| chr14:85082255:A_ATC | 14 | 85.08 | 0.94 | R | 0.70 | 0.1069 | 0.0222 | 1.43E-06 |
| chr14:85082259:C_CTT | 14 | 85.08 | 0.94 | R | 0.70 | 0.1057 | 0.0221 | 1.73E-06 |
| rs66653650 | 14 | 85.08 | 0.95 | C | 0.69 | 0.0989 | 0.0220 | 6.68E-06 |
| rs67017413 | 14 | 85.08 | 0.94 | T | 0.68 | 0.1025 | 0.0221 | 3.53E-06 |
| chr14:86917528:ACT_A | 14 | 86.92 | 0.64 | R | 0.77 | 0.1091 | 0.0280 | 9.72E-05 |
| rs74687498 | 14 | 104.85 | 0.41 | C | 0.93 | -0.2127 | 0.0545 | 9.51E-05 |
| chr15:35293366:CAT_C | 15 | 35.29 | 0.93 | R | 0.90 | 0.1332 | 0.0339 | 8.69E-05 |
| rs8026240 | 15 | 35.29 | 0.93 | T | 0.90 | 0.1347 | 0.0339 | 6.93E-05 |
| rs147080139 | 15 | 35.30 | 0.94 | A | 0.90 | 0.1347 | 0.0337 | 6.37E-05 |
| rs11855433 | 15 | 35.30 | 0.94 | A | 0.90 | 0.1346 | 0.0337 | 6.31E-05 |
| rs11855456 | 15 | 35.30 | 0.94 | A | 0.90 | 0.1347 | 0.0336 | 6.28E-05 |
| rs72702720 | 15 | 35.30 | 0.94 | C | 0.90 | 0.1347 | 0.0336 | 6.11E-05 |
| rs72702721 | 15 | 35.30 | 0.94 | G | 0.90 | 0.1347 | 0.0336 | 6.08E-05 |
| rs11852811 | 15 | 35.30 | 0.94 | G | 0.90 | 0.1348 | 0.0335 | 5.88E-05 |
| rs6495732 | 15 | 35.30 | 0.94 | G | 0.90 | 0.1345 | 0.0333 | 5.45E-05 |
| rs6495733 | 15 | 35.30 | 0.94 | G | 0.90 | 0.1343 | 0.0333 | 5.52E-05 |
| rs192006813 | 15 | 35.31 | 0.87 | A | 0.89 | 0.1342 | 0.0341 | 8.50E-05 |
| rs184152591 | 15 | 35.31 | 0.87 | G | 0.89 | 0.1340 | 0.0341 | 8.75E-05 |
| rs72702738 | 15 | 35.31 | 0.87 | C | 0.89 | 0.1340 | 0.0341 | 8.69E-05 |
| rs72702740 | 15 | 35.31 | 0.87 | A | 0.89 | 0.1341 | 0.0342 | 8.68E-05 |
| rs112080030 | 15 | 35.31 | 0.87 | G | 0.89 | 0.1345 | 0.0343 | 8.65E-05 |
| rs113948165 | 15 | 35.31 | 0.87 | A | 0.89 | 0.1345 | 0.0343 | 8.66E-05 |
| rs7164148 | 15 | 35.31 | 0.82 | A | 0.90 | 0.1411 | 0.0356 | 7.49E-05 |
| rs72702743 | 15 | 35.31 | 0.78 | T | 0.90 | 0.1465 | 0.0371 | 8.05E-05 |
| rs7166275 | 15 | 52.71 | 0.58 | G | 0.87 | -0.1445 | 0.0367 | 8.22E-05 |
| rs7168855 | 15 | 52.72 | 0.53 | T | 0.89 | -0.1630 | 0.0412 | 7.49E-05 |
| rs12325402 | 16 | 5.31 | 0.57 | C | 0.71 | -0.0963 | 0.0246 | 9.27E-05 |
| rs56174622 | 16 | 23.27 | 0.42 | G | 0.65 | -0.1122 | 0.0270 | 3.24E-05 |
| rs62031948 | 16 | 23.28 | 0.47 | T | 0.66 | -0.1061 | 0.0258 | 3.90E-05 |
| rs111645080 | 16 | 23.28 | 0.46 | C | 0.67 | -0.1088 | 0.0259 | 2.58E-05 |
| rs67622340 | 16 | 23.28 | 0.46 | G | 0.67 | -0.1114 | 0.0260 | 1.77E-05 |
| rs9934513 | 16 | 23.28 | 0.49 | T | 0.65 | -0.1033 | 0.0249 | 3.25E-05 |
| rs7200487 | 16 | 79.54 | 0.70 | G | 0.88 | -0.1404 | 0.0347 | 5.20E-05 |
| rs7204185 | 16 | 79.55 | 0.79 | A | 0.85 | -0.1250 | 0.0312 | 6.00E-05 |
| rs8052284 | 16 | 79.55 | 0.96 | T | 0.89 | -0.1223 | 0.0314 | 9.74E-05 |
| rs8051108 | 16 | 79.55 | 0.97 | C | 0.89 | -0.1221 | 0.0313 | 9.48E-05 |
| rs2549509 | 16 | 79.55 | 0.97 | T | 0.89 | -0.1226 | 0.0313 | 9.20E-05 |
| rs2549510 | 16 | 79.55 | 0.97 | A | 0.89 | -0.1225 | 0.0313 | 9.29E-05 |
| rs2720444 | 16 | 79.55 | 0.96 | A | 0.89 | -0.1228 | 0.0314 | 9.07E-05 |
| rs4888992 | 16 | 79.55 | 0.97 | T | 0.89 | -0.1224 | 0.0313 | 9.24E-05 |
| rs4888993 | 16 | 79.55 | 0.97 | T | 0.89 | -0.1224 | 0.0313 | 9.24E-05 |
| rs4888994 | 16 | 79.55 | 0.97 | G | 0.89 | -0.1224 | 0.0313 | 9.20E-05 |
| rs4888995 | 16 | 79.55 | 0.98 | A | 0.89 | -0.1215 | 0.0312 | 9.87E-05 |
| rs11150172 | 16 | 79.55 | 0.73 | C | 0.85 | -0.1247 | 0.0320 | 9.69E-05 |
| rs6503271 | 17 | 9.83 | 0.42 | G | 0.66 | 0.1415 | 0.0309 | 4.63E-06 |
| rs9302891 | 17 | 67.22 | 1.00 | G | 0.08 | 0.1654 | 0.0382 | 1.51E-05 |
| chr17:67280000:T_TA | 17 | 67.28 | 0.74 | R | 0.93 | -0.1850 | 0.0456 | 4.95E-05 |
| rs520389 | 17 | 67.30 | 0.77 | C | 0.95 | -0.1836 | 0.0468 | 8.57E-05 |
| rs72886766 | 18 | 21.94 | 0.67 | T | 0.95 | -0.2603 | 0.0541 | 1.48E-06 |
| rs192018942 | 18 | 23.20 | 0.48 | T | 0.72 | -0.1299 | 0.0298 | 1.32E-05 |
| rs4356542 | 18 | 32.79 | 0.46 | T | 0.44 | 0.1090 | 0.0279 | 9.20E-05 |
| rs113972334 | 19 | 8.11 | 0.53 | T | 0.87 | -0.1543 | 0.0386 | 6.40E-05 |
| rs34967860 | 19 | 8.11 | 0.77 | C | 0.84 | 0.1172 | 0.0295 | 7.26E-05 |
| rs74430099 | 19 | 8.12 | 0.78 | T | 0.84 | 0.1151 | 0.0292 | 8.04E-05 |
| rs148156764 | 19 | 8.12 | 0.70 | A | 0.85 | 0.1311 | 0.0319 | 3.98E-05 |
| rs141108087 | 19 | 8.12 | 0.68 | A | 0.86 | 0.1387 | 0.0334 | 3.24E-05 |
| rs11671930 | 19 | 8.12 | 0.77 | T | 0.84 | 0.1182 | 0.0293 | 5.59E-05 |
| chr19:8117424:A_AG | 19 | 8.12 | 0.77 | R | 0.84 | 0.1201 | 0.0295 | 4.61E-05 |
| rs11575008 | 19 | 8.12 | 0.77 | C | 0.84 | 0.1185 | 0.0294 | 5.41E-05 |
| rs3136654 | 19 | 8.13 | 0.74 | T | 0.77 | -0.1047 | 0.0264 | 7.21E-05 |
| rs2287937 | 19 | 8.13 | 0.77 | C | 0.78 | -0.1059 | 0.0261 | 5.04E-05 |
| rs12460243 | 19 | 8.13 | 0.78 | G | 0.78 | -0.1048 | 0.0260 | 5.65E-05 |
| rs78806879 | 19 | 8.13 | 0.77 | C | 0.78 | -0.1029 | 0.0260 | 7.42E-05 |
| rs17160128 | 19 | 8.13 | 0.77 | A | 0.78 | -0.1032 | 0.0260 | 7.22E-05 |
| rs77261076 | 19 | 8.13 | 0.76 | G | 0.78 | -0.1045 | 0.0260 | 6.04E-05 |
| rs111281472 | 19 | 8.13 | 0.77 | G | 0.78 | -0.1034 | 0.0260 | 7.15E-05 |
| rs76486281 | 19 | 8.13 | 0.75 | C | 0.78 | -0.1047 | 0.0264 | 7.28E-05 |
| rs78309858 | 19 | 8.13 | 0.76 | C | 0.78 | -0.1016 | 0.0261 | 9.71E-05 |
| rs11084697 | 19 | 33.52 | 0.62 | G | 0.74 | 0.1159 | 0.0282 | 3.96E-05 |
| chr19:33628009:TTTC_ | 19 | 33.63 | 0.74 | R | 0.63 | 0.0957 | 0.0229 | 2.84E-05 |
| chr19:33628015:CTTCT | 19 | 33.63 | 0.73 | R | 0.63 | 0.0964 | 0.0230 | 2.77E-05 |
| chr19:33629574:CTTTC | 19 | 33.63 | 0.63 | R | 0.68 | 0.1050 | 0.0262 | 6.02E-05 |
| rs884493 | 19 | 56.58 | 0.43 | T | 0.85 | -0.1759 | 0.0396 | 9.02E-06 |
| rs3746660 | 20 | 3.85 | 0.99 | C | 0.60 | -0.0821 | 0.0203 | 5.22E-05 |
| rs8126207 | 20 | 3.85 | 1.00 | G | 0.60 | -0.0824 | 0.0203 | 4.74E-05 |
| rs6515831 | 20 | 3.85 | 0.99 | T | 0.60 | -0.0857 | 0.0202 | 2.29E-05 |
| rs2464 | 20 | 3.85 | 0.98 | C | 0.60 | -0.0863 | 0.0203 | 2.18E-05 |
| chr20:3860757:AAAAC | 20 | 3.86 | 0.91 | R | 0.62 | -0.0878 | 0.0211 | 3.23E-05 |
| chr20:3860858:GC_G | 20 | 3.86 | 0.97 | R | 0.60 | -0.0860 | 0.0205 | 2.69E-05 |
| rs4813648 | 20 | 3.87 | 0.97 | C | 0.60 | -0.0824 | 0.0205 | 5.62E-05 |
| rs8117928 | 20 | 3.87 | 0.97 | G | 0.60 | -0.0824 | 0.0205 | 5.69E-05 |
| rs4813649 | 20 | 3.87 | 0.95 | C | 0.57 | -0.0801 | 0.0202 | 7.54E-05 |
| rs6076566 | 20 | 3.87 | 0.85 | C | 0.59 | -0.0876 | 0.0214 | 4.13E-05 |
| rs6116081 | 20 | 3.87 | 0.95 | G | 0.50 | 0.0845 | 0.0205 | 3.90E-05 |
| rs6037685 | 20 | 3.88 | 0.97 | A | 0.50 | 0.0847 | 0.0204 | 3.44E-05 |
| rs8117877 | 20 | 3.88 | 0.88 | C | 0.47 | 0.0859 | 0.0216 | 6.81E-05 |
| rs4815623 | 20 | 3.88 | 0.97 | T | 0.50 | 0.0821 | 0.0204 | 5.65E-05 |
| rs4815624 | 20 | 3.88 | 0.97 | T | 0.50 | 0.0821 | 0.0204 | 5.65E-05 |
| rs6052157 | 20 | 3.88 | 0.97 | G | 0.50 | 0.0820 | 0.0204 | 5.66E-05 |
| rs4815625 | 20 | 3.89 | 0.98 | G | 0.50 | 0.0835 | 0.0203 | 4.03E-05 |
| rs4815626 | 20 | 3.89 | 0.95 | C | 0.57 | -0.0794 | 0.0202 | 8.33E-05 |
| rs6052161 | 20 | 3.89 | 0.98 | C | 0.50 | 0.0835 | 0.0203 | 4.02E-05 |
| rs6052162 | 20 | 3.89 | 0.98 | C | 0.50 | 0.0837 | 0.0203 | 3.80E-05 |
| rs4813650 | 20 | 3.89 | 0.88 | C | 0.47 | 0.0888 | 0.0212 | 2.75E-05 |
| rs6052163 | 20 | 3.89 | 0.94 | A | 0.57 | -0.0795 | 0.0202 | 8.55E-05 |
| rs6037689 | 20 | 3.89 | 0.98 | A | 0.50 | 0.0837 | 0.0203 | 3.79E-05 |
| rs6052164 | 20 | 3.89 | 0.95 | A | 0.57 | -0.0795 | 0.0202 | 7.97E-05 |
| rs6052166 | 20 | 3.89 | 0.98 | A | 0.50 | 0.0836 | 0.0203 | 3.80E-05 |
| rs6139232 | 20 | 3.89 | 0.98 | T | 0.50 | 0.0836 | 0.0203 | 3.78E-05 |
| rs6139233 | 20 | 3.89 | 0.98 | T | 0.50 | 0.0835 | 0.0203 | 3.86E-05 |
| rs6052167 | 20 | 3.89 | 0.97 | C | 0.50 | 0.0836 | 0.0203 | 3.88E-05 |
| rs4815627 | 20 | 3.89 | 0.97 | C | 0.49 | 0.0840 | 0.0204 | 3.82E-05 |
| rs4815628 | 20 | 3.89 | 0.98 | C | 0.50 | 0.0833 | 0.0203 | 3.92E-05 |
| rs6139234 | 20 | 3.89 | 0.86 | T | 0.48 | 0.0860 | 0.0217 | 7.35E-05 |
| rs6139235 | 20 | 3.89 | 0.86 | C | 0.48 | 0.0860 | 0.0217 | 7.34E-05 |
| chr20:3895882:GTTTA | 20 | 3.90 | 0.99 | D | 0.50 | 0.0843 | 0.0203 | 3.37E-05 |
| rs6052169 | 20 | 3.90 | 1.00 | T | 0.50 | 0.0840 | 0.0203 | 3.42E-05 |
| rs241595 | 20 | 3.90 | 0.99 | G | 0.51 | 0.0843 | 0.0203 | 3.35E-05 |
| rs241596 | 20 | 3.91 | 0.96 | G | 0.50 | 0.0857 | 0.0205 | 2.86E-05 |
| rs241598 | 20 | 3.91 | 0.88 | G | 0.55 | 0.0871 | 0.0216 | 5.57E-05 |
| rs241599 | 20 | 3.91 | 0.97 | G | 0.51 | 0.0841 | 0.0205 | 4.08E-05 |
| rs241600 | 20 | 3.91 | 1.00 | T | 0.50 | 0.0806 | 0.0202 | 6.70E-05 |
| rs241601 | 20 | 3.91 | 1.00 | A | 0.50 | 0.0806 | 0.0202 | 6.72E-05 |
| rs241602 | 20 | 3.91 | 0.93 | C | 0.48 | 0.0820 | 0.0209 | 8.68E-05 |
| rs241603 | 20 | 3.91 | 0.93 | G | 0.48 | 0.0815 | 0.0209 | 9.26E-05 |
| rs241604 | 20 | 3.91 | 1.00 | G | 0.50 | 0.0806 | 0.0203 | 7.18E-05 |
| rs241606 | 20 | 3.92 | 1.00 | A | 0.50 | 0.0804 | 0.0202 | 6.96E-05 |
| rs47794 | 20 | 3.92 | 0.95 | A | 0.52 | 0.0834 | 0.0207 | 5.43E-05 |
| rs241617 | 20 | 3.92 | 1.00 | A | 0.50 | 0.0803 | 0.0202 | 7.15E-05 |
| rs241624 | 20 | 3.92 | 1.00 | G | 0.51 | 0.0821 | 0.0202 | 5.03E-05 |
| rs241642 | 20 | 3.93 | 0.98 | G | 0.51 | 0.0855 | 0.0205 | 3.05E-05 |
| rs6123356 | 20 | 52.69 | 0.94 | A | 0.89 | -0.1357 | 0.0314 | 1.52E-05 |
| rs73130100 | 20 | 52.69 | 0.99 | A | 0.89 | -0.1396 | 0.0310 | 6.63E-06 |
| rs399448 | 20 | 52.69 | 0.95 | A | 0.86 | -0.1330 | 0.0279 | 1.93E-06 |
| rs6127088 | 20 | 52.69 | 0.98 | C | 0.88 | -0.1359 | 0.0298 | 5.23E-06 |
| rs6127089 | 20 | 52.69 | 0.99 | G | 0.89 | -0.1401 | 0.0309 | 5.79E-06 |
| rs412429 | 20 | 52.69 | 0.95 | T | 0.86 | -0.1333 | 0.0279 | 1.80E-06 |
| rs6127090 | 20 | 52.69 | 0.99 | T | 0.89 | -0.1403 | 0.0309 | 5.68E-06 |
| rs12625949 | 20 | 52.70 | 0.96 | C | 0.88 | -0.1384 | 0.0305 | 5.67E-06 |
| chr20:52696975:AT_A | 20 | 52.70 | 0.92 | R | 0.88 | -0.1405 | 0.0304 | 3.93E-06 |
| rs1543703 | 20 | 52.70 | 0.98 | T | 0.34 | 0.0818 | 0.0209 | 9.36E-05 |
| rs13041834 | 20 | 52.70 | 0.96 | T | 0.34 | 0.0833 | 0.0212 | 8.62E-05 |
| chr20:52928609:GTCTC | 20 | 52.93 | 0.92 | R | 0.95 | -0.1862 | 0.0450 | 3.47E-05 |
| rs67043187 | 20 | 52.93 | 0.92 | T | 0.95 | -0.1864 | 0.0450 | 3.45E-05 |
| rs67291713 | 20 | 52.93 | 0.92 | C | 0.95 | -0.1865 | 0.0450 | 3.44E-05 |
| rs67348261 | 20 | 52.93 | 0.92 | A | 0.95 | -0.1869 | 0.0450 | 3.33E-05 |
| rs16999389 | 20 | 52.93 | 0.92 | G | 0.95 | -0.1866 | 0.0451 | 3.45E-05 |
| chr20:52929573:C_CTA | 20 | 52.93 | 0.91 | R | 0.95 | -0.1866 | 0.0451 | 3.48E-05 |
| rs60650892 | 20 | 52.93 | 0.91 | T | 0.95 | -0.1868 | 0.0451 | 3.47E-05 |
| rs60900910 | 20 | 52.93 | 0.91 | A | 0.95 | -0.1868 | 0.0451 | 3.48E-05 |
| rs6097909 | 20 | 52.93 | 0.91 | G | 0.95 | -0.1870 | 0.0452 | 3.49E-05 |
| rs16999393 | 20 | 52.93 | 0.91 | G | 0.95 | -0.1870 | 0.0452 | 3.50E-05 |
| rs6097910 | 20 | 52.93 | 0.88 | T | 0.95 | -0.1873 | 0.0453 | 3.49E-05 |
| rs6097911 | 20 | 52.93 | 0.88 | C | 0.95 | -0.1873 | 0.0453 | 3.50E-05 |
| rs6097912 | 20 | 52.93 | 0.88 | G | 0.95 | -0.1874 | 0.0453 | 3.50E-05 |
| rs67738640 | 20 | 52.93 | 0.87 | T | 0.95 | -0.1875 | 0.0453 | 3.51E-05 |
| chr20:52940470:G_GT | 20 | 52.94 | 0.57 | R | 0.93 | -0.2015 | 0.0499 | 5.33E-05 |
| rs28579934 | 20 | 52.94 | 0.80 | C | 0.95 | -0.1908 | 0.0486 | 8.67E-05 |
| rs9980736 | 21 | 20.98 | 0.82 | T | 0.84 | -0.1101 | 0.0280 | 8.37E-05 |
| rs9981107 | 21 | 20.99 | 0.81 | G | 0.82 | -0.1078 | 0.0263 | 4.05E-05 |
| rs2825678 | 21 | 20.99 | 0.81 | A | 0.82 | -0.1109 | 0.0262 | 2.28E-05 |
| rs55702233 | 21 | 20.99 | 0.81 | A | 0.82 | -0.1109 | 0.0262 | 2.27E-05 |
| rs7281590 | 21 | 20.99 | 0.80 | G | 0.81 | -0.1105 | 0.0261 | 2.30E-05 |
| rs9977238 | 21 | 20.99 | 0.81 | T | 0.82 | -0.1115 | 0.0263 | 2.31E-05 |
| rs2825680 | 21 | 20.99 | 0.81 | G | 0.80 | -0.1079 | 0.0258 | 2.82E-05 |
| rs2825681 | 21 | 20.99 | 0.81 | A | 0.80 | -0.1073 | 0.0258 | 3.11E-05 |
| rs2825682 | 21 | 20.99 | 1.00 | A | 0.17 | 0.1243 | 0.0262 | 2.02E-06 |
| chr21:20991988:A_AAA | 21 | 20.99 | 0.81 | R | 0.79 | -0.1046 | 0.0248 | 2.43E-05 |
| rs2825683 | 21 | 20.99 | 0.82 | C | 0.79 | -0.1028 | 0.0248 | 3.49E-05 |
| chr21:20993953:CAGAA | 21 | 20.99 | 0.82 | R | 0.79 | -0.1020 | 0.0248 | 3.86E-05 |
| rs8132923 | 21 | 20.99 | 0.83 | T | 0.79 | -0.1017 | 0.0247 | 3.79E-05 |
| rs8129479 | 21 | 20.99 | 0.83 | A | 0.79 | -0.1016 | 0.0247 | 3.82E-05 |
| rs11908861 | 21 | 21.00 | 0.83 | A | 0.79 | -0.1017 | 0.0247 | 3.93E-05 |
| rs2825685 | 21 | 21.00 | 0.80 | T | 0.81 | -0.1079 | 0.0259 | 3.02E-05 |
| rs7277887 | 21 | 21.00 | 0.81 | G | 0.81 | -0.1076 | 0.0259 | 3.15E-05 |
| rs2825686 | 21 | 21.00 | 0.84 | C | 0.79 | -0.1002 | 0.0247 | 4.82E-05 |
| rs9983677 | 21 | 21.00 | 0.85 | T | 0.79 | -0.0998 | 0.0246 | 5.01E-05 |
| chr21:20998203:A_AG | 21 | 21.00 | 0.84 | R | 0.79 | -0.0999 | 0.0247 | 5.21E-05 |
| chr21:20998487:AAC_A | 21 | 21.00 | 0.81 | R | 0.81 | -0.1027 | 0.0256 | 6.02E-05 |
| rs2825689 | 21 | 21.00 | 0.81 | T | 0.81 | -0.1070 | 0.0259 | 3.56E-05 |
| chr21:21000453:AAAAA | 21 | 21.00 | 0.81 | R | 0.81 | -0.1050 | 0.0258 | 4.67E-05 |
| rs11908784 | 21 | 21.00 | 0.84 | A | 0.79 | -0.0971 | 0.0246 | 7.66E-05 |
| rs67165434 | 21 | 21.00 | 0.79 | T | 0.84 | -0.1175 | 0.0290 | 4.92E-05 |
| rs2006900 | 21 | 21.00 | 0.84 | A | 0.79 | -0.0983 | 0.0247 | 6.75E-05 |
| rs11909936 | 21 | 21.00 | 0.85 | T | 0.79 | -0.0968 | 0.0247 | 8.82E-05 |
| rs67933344 | 21 | 21.01 | 0.81 | C | 0.81 | -0.1044 | 0.0259 | 5.70E-05 |
| chr21:21007729:CAT_C | 21 | 21.01 | 0.82 | R | 0.81 | -0.1034 | 0.0259 | 6.73E-05 |
| rs2825693 | 21 | 21.01 | 0.87 | T | 0.79 | -0.0961 | 0.0247 | 9.67E-05 |
| rs2825694 | 21 | 21.01 | 0.83 | A | 0.81 | -0.1039 | 0.0259 | 5.96E-05 |
| rs2154623 | 21 | 21.01 | 0.82 | T | 0.81 | -0.1040 | 0.0260 | 6.37E-05 |
| rs2825696 | 21 | 21.01 | 0.87 | A | 0.79 | -0.0959 | 0.0246 | 9.65E-05 |
| rs2825697 | 21 | 21.01 | 0.82 | C | 0.81 | -0.1044 | 0.0261 | 6.18E-05 |
| rs2825698 | 21 | 21.01 | 0.85 | A | 0.79 | -0.0966 | 0.0247 | 9.34E-05 |
| rs2825699 | 21 | 21.01 | 0.82 | A | 0.81 | -0.1047 | 0.0261 | 6.12E-05 |
| rs2825700 | 21 | 21.01 | 0.82 | C | 0.81 | -0.1041 | 0.0261 | 6.42E-05 |
| rs55833243 | 21 | 21.01 | 0.80 | A | 0.81 | -0.1095 | 0.0271 | 5.22E-05 |
| rs67359427 | 21 | 21.01 | 0.81 | A | 0.81 | -0.1088 | 0.0273 | 6.61E-05 |
| rs2825701 | 21 | 21.01 | 0.86 | T | 0.79 | -0.1004 | 0.0258 | 9.82E-05 |
| rs67677722 | 21 | 21.01 | 0.81 | C | 0.81 | -0.1089 | 0.0273 | 6.56E-05 |
| rs112510755 | 21 | 21.02 | 0.81 | G | 0.82 | -0.1094 | 0.0274 | 6.38E-05 |
| rs73322363 | 21 | 21.02 | 0.81 | C | 0.82 | -0.1094 | 0.0274 | 6.37E-05 |
| rs9984418 | 21 | 21.02 | 0.85 | T | 0.80 | -0.1026 | 0.0262 | 8.91E-05 |
| rs9981114 | 21 | 21.02 | 0.85 | G | 0.80 | -0.1026 | 0.0262 | 8.89E-05 |
| rs2825702 | 21 | 21.02 | 0.82 | A | 0.81 | -0.1095 | 0.0274 | 6.35E-05 |
| rs2825703 | 21 | 21.02 | 0.79 | A | 0.81 | -0.1085 | 0.0275 | 8.08E-05 |
| chr21:21018613:AAG_A | 21 | 21.02 | 0.79 | R | 0.82 | -0.1134 | 0.0289 | 8.74E-05 |
| rs2825707 | 21 | 21.02 | 0.75 | A | 0.81 | -0.1163 | 0.0287 | 5.03E-05 |
| rs2825708 | 21 | 21.02 | 0.80 | A | 0.79 | -0.1054 | 0.0270 | 9.34E-05 |
| rs2825709 | 21 | 21.02 | 0.79 | T | 0.79 | -0.1077 | 0.0273 | 8.09E-05 |
| rs8129559 | 21 | 21.92 | 0.85 | A | 0.75 | -0.0934 | 0.0235 | 7.06E-05 |
| rs58668017 | 21 | 36.08 | 0.89 | T | 0.93 | -0.1684 | 0.0411 | 4.21E-05 |
| rs59160867 | 21 | 36.08 | 0.89 | C | 0.93 | -0.1698 | 0.0411 | 3.63E-05 |
| chr21:36086993:A_AC | 21 | 36.09 | 0.88 | R | 0.93 | -0.1666 | 0.0407 | 4.23E-05 |
| chr21:36087005:A_AC | 21 | 36.09 | 0.87 | R | 0.93 | -0.1660 | 0.0407 | 4.50E-05 |
| rs2834601 | 21 | 36.09 | 1.00 | T | 0.07 | 0.1578 | 0.0404 | 9.35E-05 |
| chr21:36090536:G_GTT | 21 | 36.09 | 0.84 | R | 0.93 | -0.1633 | 0.0410 | 6.72E-05 |
| rs113759928 | 21 | 36.09 | 0.84 | C | 0.93 | -0.1626 | 0.0410 | 7.19E-05 |
| rs111498760 | 21 | 36.09 | 0.84 | T | 0.93 | -0.1627 | 0.0411 | 7.59E-05 |
| rs2834603 | 21 | 36.09 | 0.83 | A | 0.93 | -0.1630 | 0.0411 | 7.28E-05 |
| rs9604779 | 22 | 18.12 | 0.83 | T | 0.94 | 0.1719 | 0.0435 | 7.74E-05 |
| rs9605357 | 22 | 18.12 | 0.81 | C | 0.93 | 0.1694 | 0.0428 | 7.62E-05 |
| rs5992775 | 22 | 18.12 | 0.81 | G | 0.93 | 0.1707 | 0.0430 | 7.11E-05 |
| rs67786863 | 22 | 18.12 | 0.82 | R | 0.93 | 0.1711 | 0.0431 | 7.21E-05 |
| chr22:18124153:A_AT | 22 | 18.12 | 0.77 | R | 0.92 | 0.1688 | 0.0420 | 5.86E-05 |
| rs12166769 | 22 | 18.13 | 0.93 | G | 0.93 | 0.1625 | 0.0402 | 5.42E-05 |
| rs9605366 | 22 | 18.13 | 0.93 | C | 0.93 | 0.1623 | 0.0402 | 5.46E-05 |
| rs9604782 | 22 | 18.13 | 0.91 | T | 0.93 | 0.1728 | 0.0412 | 2.75E-05 |
| rs9605367 | 22 | 18.13 | 0.94 | T | 0.93 | 0.1613 | 0.0401 | 5.66E-05 |
| rs5992778 | 22 | 18.13 | 0.96 | A | 0.93 | 0.1590 | 0.0396 | 6.04E-05 |
| rs9618087 | 22 | 18.14 | 0.96 | A | 0.93 | 0.1579 | 0.0395 | 6.44E-05 |
| rs66927820 | 22 | 18.14 | 0.92 | R | 0.93 | 0.1559 | 0.0396 | 8.16E-05 |
| rs5992782 | 22 | 18.15 | 0.97 | C | 0.93 | 0.1576 | 0.0394 | 6.35E-05 |
| rs5992783 | 22 | 18.15 | 0.97 | G | 0.93 | 0.1583 | 0.0395 | 6.24E-05 |
| rs5992784 | 22 | 18.15 | 0.97 | A | 0.93 | 0.1582 | 0.0395 | 6.24E-05 |
| rs12170245 | 22 | 18.15 | 0.97 | C | 0.93 | 0.1573 | 0.0394 | 6.40E-05 |
| rs9604784 | 22 | 18.16 | 0.97 | C | 0.93 | 0.1578 | 0.0395 | 6.43E-05 |
| rs5992786 | 22 | 18.16 | 0.97 | A | 0.93 | 0.1567 | 0.0393 | 6.51E-05 |
| rs5992090 | 22 | 18.16 | 0.97 | C | 0.93 | 0.1574 | 0.0394 | 6.39E-05 |
| rs9605382 | 22 | 18.16 | 0.97 | G | 0.93 | 0.1578 | 0.0396 | 6.83E-05 |
| rs5992787 | 22 | 18.16 | 0.97 | T | 0.93 | 0.1577 | 0.0395 | 6.47E-05 |
| rs5992789 | 22 | 18.17 | 0.97 | T | 0.93 | 0.1565 | 0.0392 | 6.55E-05 |
| rs9604785 | 22 | 18.17 | 0.97 | T | 0.93 | 0.1568 | 0.0393 | 6.59E-05 |
| rs5992092 | 22 | 18.17 | 0.98 | T | 0.93 | 0.1558 | 0.0391 | 6.86E-05 |
| rs5992093 | 22 | 18.18 | 0.98 | A | 0.93 | 0.1557 | 0.0391 | 6.87E-05 |
| rs5992792 | 22 | 18.18 | 0.97 | T | 0.93 | 0.1570 | 0.0391 | 6.04E-05 |
| rs12160637 | 22 | 18.18 | 0.97 | T | 0.93 | 0.1560 | 0.0392 | 6.87E-05 |
| rs5992094 | 22 | 18.18 | 0.97 | G | 0.93 | 0.1560 | 0.0392 | 6.88E-05 |
| rs5992793 | 22 | 18.18 | 0.97 | T | 0.93 | 0.1550 | 0.0392 | 7.79E-05 |
| rs5992795 | 22 | 18.19 | 0.97 | C | 0.93 | 0.1561 | 0.0392 | 6.88E-05 |
| rs9617611 | 22 | 18.19 | 0.98 | C | 0.93 | 0.1567 | 0.0389 | 5.70E-05 |
| rs5992796 | 22 | 18.19 | 0.98 | T | 0.93 | 0.1553 | 0.0391 | 7.05E-05 |
| rs5992797 | 22 | 18.19 | 0.97 | T | 0.93 | 0.1553 | 0.0391 | 7.12E-05 |
| rs5992096 | 22 | 18.19 | 0.98 | T | 0.93 | 0.1553 | 0.0391 | 7.05E-05 |
| rs2401168 | 22 | 18.19 | 0.95 | G | 0.93 | 0.1594 | 0.0399 | 6.38E-05 |
| rs5992799 | 22 | 18.19 | 0.97 | G | 0.93 | 0.1565 | 0.0394 | 7.24E-05 |
| rs5992800 | 22 | 18.19 | 0.97 | C | 0.93 | 0.1566 | 0.0394 | 6.95E-05 |
| rs5992801 | 22 | 18.19 | 0.98 | C | 0.93 | 0.1568 | 0.0389 | 5.66E-05 |
| rs12170986 | 22 | 18.20 | 0.96 | C | 0.93 | 0.1569 | 0.0390 | 5.72E-05 |
| rs5992097 | 22 | 18.20 | 0.97 | C | 0.93 | 0.1557 | 0.0392 | 6.98E-05 |
| rs9605385 | 22 | 18.20 | 0.94 | G | 0.93 | 0.1556 | 0.0396 | 8.48E-05 |
| rs5992804 | 22 | 18.20 | 0.94 | C | 0.93 | 0.1556 | 0.0394 | 7.90E-05 |
| rs58210163 | 22 | 18.20 | 0.95 | T | 0.93 | 0.1561 | 0.0391 | 6.49E-05 |
| rs6000157 | 22 | 36.56 | 0.98 | T | 0.66 | -0.0837 | 0.0204 | 4.19E-05 |

Abbreviations: Chr-Chromosome, s.e.-standard error, Rsq-imputation quality estimate.
