# Supplementary material for: Genetic Variants Modulating CRIPTO Serum Levels Identified by Genome-Wide Association Study in Cilento Isolates
Source: PLoS Genet. 2015 Jan 28;11(1):e1004976. doi: 10.1371/journal.pgen.1004976 (PMC4309561; doi:10.1371/journal.pgen.1004976)
Supplement: S2 Table — (DOCX) [file pgen.1004976.s006.docx]

**Table S2.**

|  | | | |
| --- | --- | --- | --- |
| **Chr** | **SNP** | **Region (Mb)** | **Seed genes^1^** |
| 2 | rs6739316 | 218.67-218.87 | *TNS1* |
|  |  |  | *MIR6809* |
| 3 | rs3806702 | 46.50-46.74 | *TDGF1 (CRIPTO)* |
| 9 | rs17087824 | 87.36-87.63 | *NTRK2* |
| 14 | rs74062852 | 70.56-70.79 | *SLC8A3* |
|  |  |  | *ADAM21P1* |
| 15 | rs7168855 | 52.61-52.81 | *MYO5A* |
| 17 | rs6503271 | 9.7-9.9 | *GAS7* |
|  |  |  | *RCVRN* |
|  |  |  | *GLP2R* |
| 1 RefSeq Genes from UCSC Genome Browser | | | |
