# Supplementary material for: Genetic Variants Modulating CRIPTO Serum Levels Identified by Genome-Wide Association Study in Cilento Isolates
Source: PLoS Genet. 2015 Jan 28;11(1):e1004976. doi: 10.1371/journal.pgen.1004976 (PMC4309561; doi:10.1371/journal.pgen.1004976)
Supplement: S3 Table — (DOCX) [file pgen.1004976.s007.docx]

**Table S3.**

| **Function** | **P-value** | **Molecules** |
| --- | --- | --- |
| Cell migration | 4.39*10^-13^ | CCL20, CCL22, CCL26, CCL3, CCL5, CD2, CXCR4, ERBB4, ERK1/2, F2, Fcer1, GSK3B, HPSE, IL13, IL3, IL33, Igm, KITLG, LEP, PDLIM2, PLD1, PPARG, PRKCD, Rock, SOCS1, SRC(family), TDGF1, TNS1, TSLP, VEGF |
| Proliferation of tumor cells | 2.01*10^-09^ | CCL3, CXCR4, ERBB4, ERK1/2, F2, GSK3B, HPSE, IL13, IL3, KITLG, LEP, PLD1, PPARG, PRKCD, SOCS1, SRC(family), TDGF1, VEGF |
| Differentiation of cells | 3.10*10^-09^ | BAD, CCL3, CCL5, CD2, CXCR4, ERBB4, ERK1/2, F2, GAS7, GSK3B, HPSE, IL13, IL3, IL33, KITLG, LEP, PPARG, PRKCD, Rock, SNCA, SOCS1, SRC(family), TDGF1, TSLP, VEGF |
| Blood vessel development | 6.88*10^-08^ | CCL26, CCL5, CXCR4, ERK1/2, F2, GSK3B, HPSE, IL13, IL3, KITLG, LEP, PLD1, PPARG, PRKCD, SRC(family), TDGF1, VEGF |
